# Biosensor that Detects Stress Caused by Periplasmic Proteins

**DOI:** 10.1021/acssynbio.3c00720

**Published:** 2024-04-27

**Authors:** Alister J. Cumming, Diana Khananisho, Mateusz Balka, Nicklas Liljestrand, Daniel O. Daley

**Affiliations:** Department of Biochemistry and Biophysics, Stockholm University, Stockholm SE-19468, Sweden

**Keywords:** biosensor, recombinant protein production, periplasm, heat shock response, envelope stress response, ibpA, cpx

## Abstract

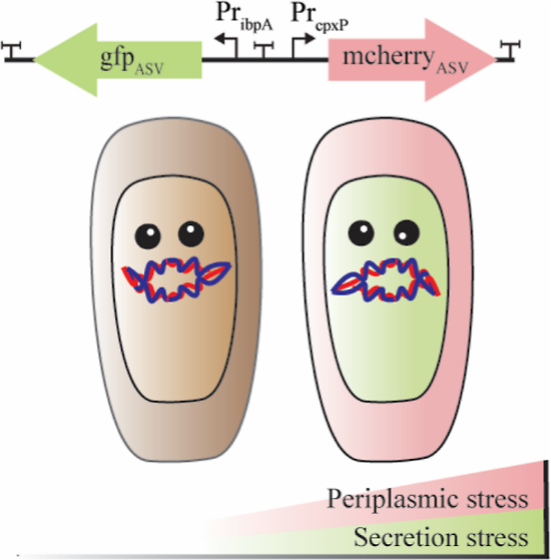

*Escherichia coli* is often used as a factory to produce recombinant proteins. In many cases, the recombinant protein needs disulfide bonds to fold and function correctly. These proteins are genetically fused to a signal peptide so that they are secreted to the oxidizing environment of the periplasm (where the enzymes required for disulfide bond formation exist). Currently, it is difficult to determine *in vivo* whether a recombinant protein is efficiently secreted from the cytoplasm and folded in the periplasm or if there is a bottleneck in one of these steps because cellular capacity has been exceeded. To address this problem, we have developed a biosensor that detects cellular stress caused by (1) inefficient secretion of proteins from the cytoplasm and (2) aggregation of proteins in the periplasm. We demonstrate how the fluorescence fingerprint obtained from the biosensor can be used to identify induction conditions that do not exceed the capacity of the cell and therefore do not cause cellular stress. These induction conditions result in more effective biomass and in some cases higher titers of soluble recombinant proteins.

## Introduction

Recombinant antibody fragments, hormones, and enzymes are often targeted to the periplasm of *Escherichia coli*,^1−4^ as it contains enzymes required for the formation of disulfide bonds.^5−7^ The periplasm also offers the possibility to avoid cytoplasmic proteases, to control the identity of the N-terminal amino acid, and to simplify downstream purification processes.^8^

A recombinant protein is targeted to the periplasm by fusing it with a signal peptide. The signal peptide delays folding^9−11^ and guides the recombinant protein to the Sec translocon so that it can be secreted across the cytoplasmic membrane.^8,12^ Secretion is facilitated by cytoplasmic chaperones, such as Trigger factor and SecB, and auxiliary modules at the Sec translocon, such as SecA, SecDFYajC, and PpiD.^8,13^ As the signal peptide is hydrophobic, it anchors the recombinant protein in the cytoplasmic membrane, until the signal peptide is cleaved by the signal peptidase LepB (on the periplasmic side).^14^ Thus, only the mature protein is released to the periplasm, where it folds with the assistance of a network of periplasmic chaperones, disulfide catalysts, and isomerases.^15,16^ It is also possible to target a recombinant protein to the periplasm, by fusing it to a signal peptide that guides it to the Tat translocon.^17,18^ A Tat signal peptide allows the recombinant protein to be folded in the cytoplasm and then translocated to the periplasm.

Targeting a recombinant protein to the periplasm is a challenging endeavor. If the expression levels exceed the capacity of the Sec translocon, the recombinant protein will accumulate in the cytoplasm.^4,8,16,19,20^ This will result in a heat shock response, impaired energy metabolism, and reduced cell growth (i.e., biomass).^20−22^ If the expression levels exceed the chaperone capacity in the periplasm, the recombinant protein may misfold and/or aggregate.^8^ This will result in an envelope stress response and potentially cell death.^23^ Maximizing production titers for periplasmic proteins is therefore a tedious process of optimizing expression conditions (inducer concentration, induction time, media, and temperature) so that the capacity of the Sec translocon and the periplasmic chaperone network is not exceeded.^4,19,24^

Currently, expression conditions are evaluated using classical biochemical approaches or variants thereof. For example, inefficient secretion to the periplasm is detected by sodium dodecyl sulfate-polyacrylamide gel electrophoresis (SDS-PAGE) and Western blotting to determine if the signal peptide is present (indicative of a cytoplasmic location) or absent (indicative of a periplasmic location). Misfolding/aggregation is detected by cell lysis, fractionation into soluble and insoluble fractions, and then SDS-PAGE and Western blotting.^25^ These methods are time-consuming and are not compatible with single-cell screening by fluorescence-activated cell sorting or other high-throughput screening approaches.

Herein, we have engineered a dual color biosensor that detects stress caused by (1) the inefficient secretion of proteins from the cytoplasm and (2) misfolding/aggregation of proteins in the periplasm. We demonstrate how the fluorescence fingerprint obtained from the genetic sensor can be used to identify induction conditions that do not exceed the capacity of the cell and therefore do not cause cellular stress. We also highlight some limitations of the approach.

## Results

### Maltose-Binding Protein as a Model System for Secretion and Folding

Maltose-binding protein (MalE) is an *E. coli* protein that binds maltose and maltodextrins in the periplasm and delivers them to an ABC transporter in the inner membrane consisting of MalFGK_2_.^26^ It has been extensively used as a model system to study protein secretion and folding in the periplasm (refs (9,10,23,27−32) and references therein). This body of work has established that the protein is synthesized with an N-terminal signal peptide (preMalE) and then post-translationally trafficked across the inner membrane by the Sec translocon. Once in the periplasm, the signal peptide is removed, and MalE is folded.^33,34^ The final structure is an ellipsoid consisting of two similar globular domains with a deep substrate-binding pocket.^35^ Mutations in MalE cause it to aggregate in the periplasm. In this study, we have used preMalEΔC (lacking the C-terminal 94 amino acids) and preMalE31 (Gly-32-Asp and Ile-33-Pro).^23^ Finally, preMalE, preMalEΔC, and preMalE31 can be engineered so that they are retained in the cytoplasm by removing the native MalE signal peptide (i.e., ΔssMalE, ΔssMalEΔC, and ΔssMalE31).

**Figure 1:**
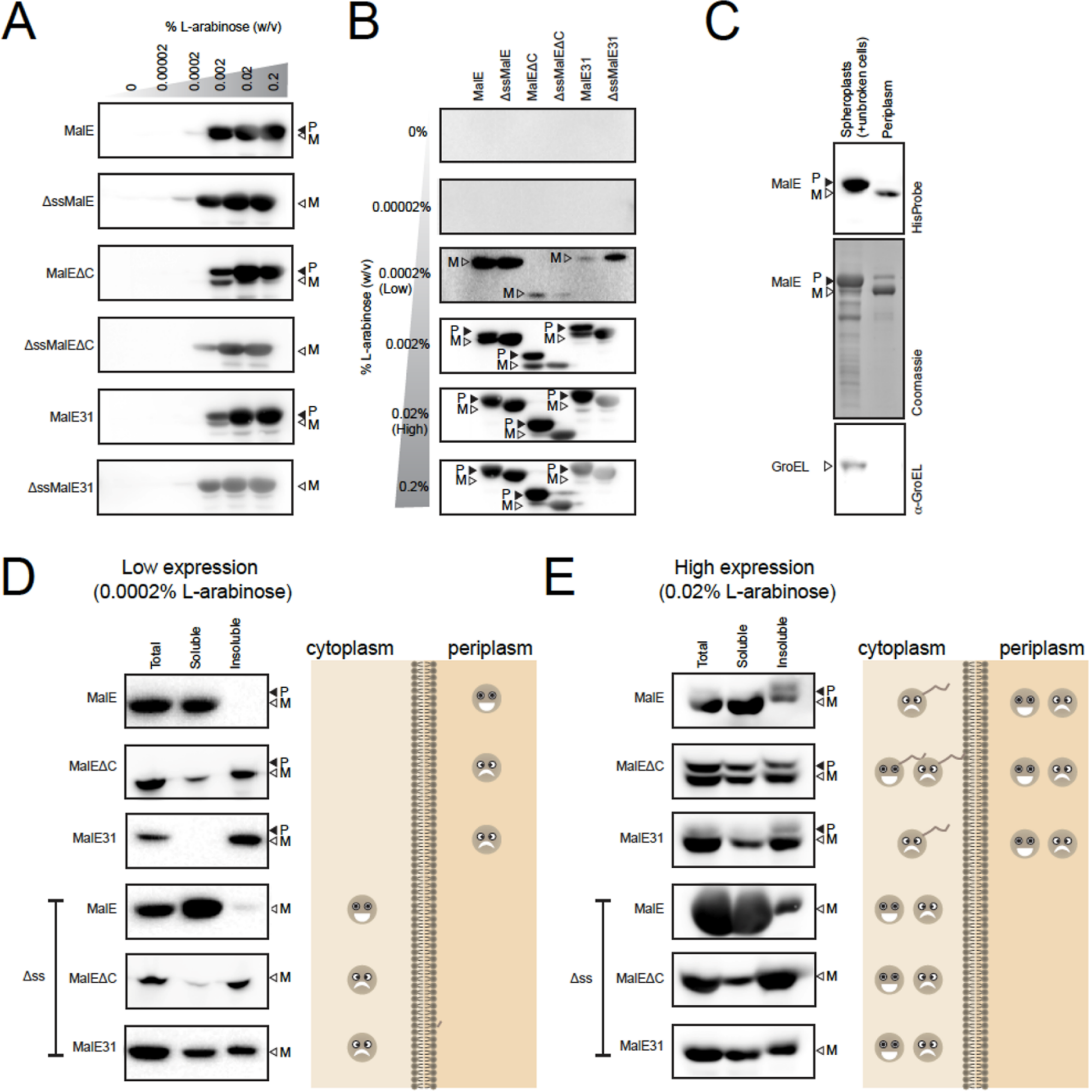
Maltose-binding protein (MalE/MBP) and misfolding variants can be used as model systems for protein secretion and folding. (A) Western blots of preMalE, ΔssMalE, preMalEΔC, ΔssMalEΔC, preMalE31, and ΔssMalE31 were expressed with increasing concentrations of l-arabinose. A 50 μL aliquot of each culture was separated by SDS-PAGE. MalE was detected by Western blotting with a HisProbe-HRP conjugate. The mature versions (periplasmic localization) are denoted M, and the signal sequence-containing versions (cytoplasmic localization) are denoted P. (B) As per (A), except that samples induced with the same concentration of l-arabinose were separated on the same gel so that the presence or absence of the signal sequence could be more easily visualized. The mature form of the protein was identified by comparing the mobility in SDS-PAGE to versions where the signal peptide was not included (ΔssMalE, ΔssMalEΔC, and ΔssMalE31). The mature versions (periplasmic localization) are denoted M, and the signal sequence-containing versions (cytoplasmic localization) are denoted P. (C) preMalE was induced with 0.02% (w/v) l-arabinose (high expression), and the localization of preMalE and MalE was assessed by fractionating the periplasm from spheroplasts (and unbroken cells). Samples were analyzed by SDS-PAGE and Western blotting with a HisProbe-HRP conjugate (top). The signal sequence-containing version of the protein is denoted P, and the mature form is denoted M. Samples were also analyzed by Coomassie staining (middle) and by Western blotting with antisera to GroEL (a cytoplasmic marker, bottom). The experiment confirms that only the mature form is in the periplasmic fraction. (D) Left, preMalE, preMalEΔC, preMalE31, ΔssMalE, ΔssMalEΔ*C,* and ΔssMalE31 were induced with 0.0002% (w/v) l-arabinose (low expression), and solubility was assessed by cell lysis, followed by fractionation into soluble and insoluble fractions by centrifugation. The total lysate, soluble, and insoluble fractions were analyzed by SDS-PAGE and Western blotting with a HisProbe-HRP conjugate. The signal sequence-containing version of the protein is denoted P, and the mature form is denoted M. For preMalE, preMalEΔC, and preMalE31, the signal sequence was removed, indicating that they were efficiently translocated to the periplasm (see B,C). Periplasmic MalE was soluble; MalEΔ*C* was partly soluble and partly insoluble; and MalE31 was insoluble. ΔssMalE, ΔssMalEΔC, and ΔssMalE31 lack a signal peptide and remain in the cytoplasm. Cytoplasmic MalE was soluble, MalEΔ*C* was insoluble, and MalE31 was partly soluble and partly insoluble. Right, cartoon representation of the data. (E) As per (D), except that proteins were induced with 0.02% (w/v) l-arabinose (high expression). All three proteins are partially retained in the cytoplasm, with the signal peptide still attached. They are also partially secreted to the periplasm, where there is a soluble and an insoluble population.

Herein, the coding sequences for preMalE, preMalEΔC, preMalE31, ΔssMalE, ΔssMalEΔC, and ΔssMalE31 were cloned into the pBAD expression plasmid and expressed by induction with l-arabinose in the MC1061 strain (which cannot metabolize l-arabinose). The cells were grown in 5 mL of LB media incubated in a 24-well plate at 37 °C with shaking. By an increase in the l-arabinose concentration, it was possible to titrate the expression of the proteins (Figure 1A). It was also possible to monitor the efficiency of secretion to the periplasm by monitoring the removal of the native MalE signal peptide by SDS-PAGE (Figure 1B). The presence of the signal peptide is a widely acknowledged indication that the protein is in the cytoplasm, and its removal indicates that the protein is in the periplasm.^14^ We confirmed this by fractionating the periplasm and monitoring the localization of preMalE and MalE. These data show that only MalE was in the periplasm. preMalE was retained in the fraction containing spheroplasts (and unbroken cells) (Figure 1C). The solubility of the different MalE variants was confirmed by fractionating cells into soluble and insoluble fractions (Figure 1D,E). These observations are consistent with published work.^30,32,36,37^

Taken together, the data indicate that preMalE was efficiently secreted to the periplasm at low levels of expression (i.e., induction with 0.0002% (w/v) l-arabinose), as the signal peptide was removed (Figure 1B). Moreover, the mature domain (MalE) was soluble in the periplasm (Figure 1D). At high levels of expression (i.e., induction with 0.02% (w/v) l-arabinose), the signal peptide was detectable, indicating that a proportion of preMalE was retained in the cytoplasm and the capacity of the Sec translocon was exceeded. preMalE in the cytoplasm was insoluble (Figure 1E). At high levels of expression, the mature domain (MalE) was also detected. Since it lacks the signal peptide, it was judged to be in the periplasm. This population was partly soluble and partly insoluble (Figure 1E). preMalE^ΔC^ and preMalE31 were also efficiently secreted to the periplasm at low levels but not at high levels of expression (Figure 1B). Both were partially soluble and partly insoluble (Figure 1D,E).

### Stress Caused by Inefficient Secretion from the Cytoplasm can be Detected Using [P_ibpA_-gfp_ASV_]

**Figure 2:**
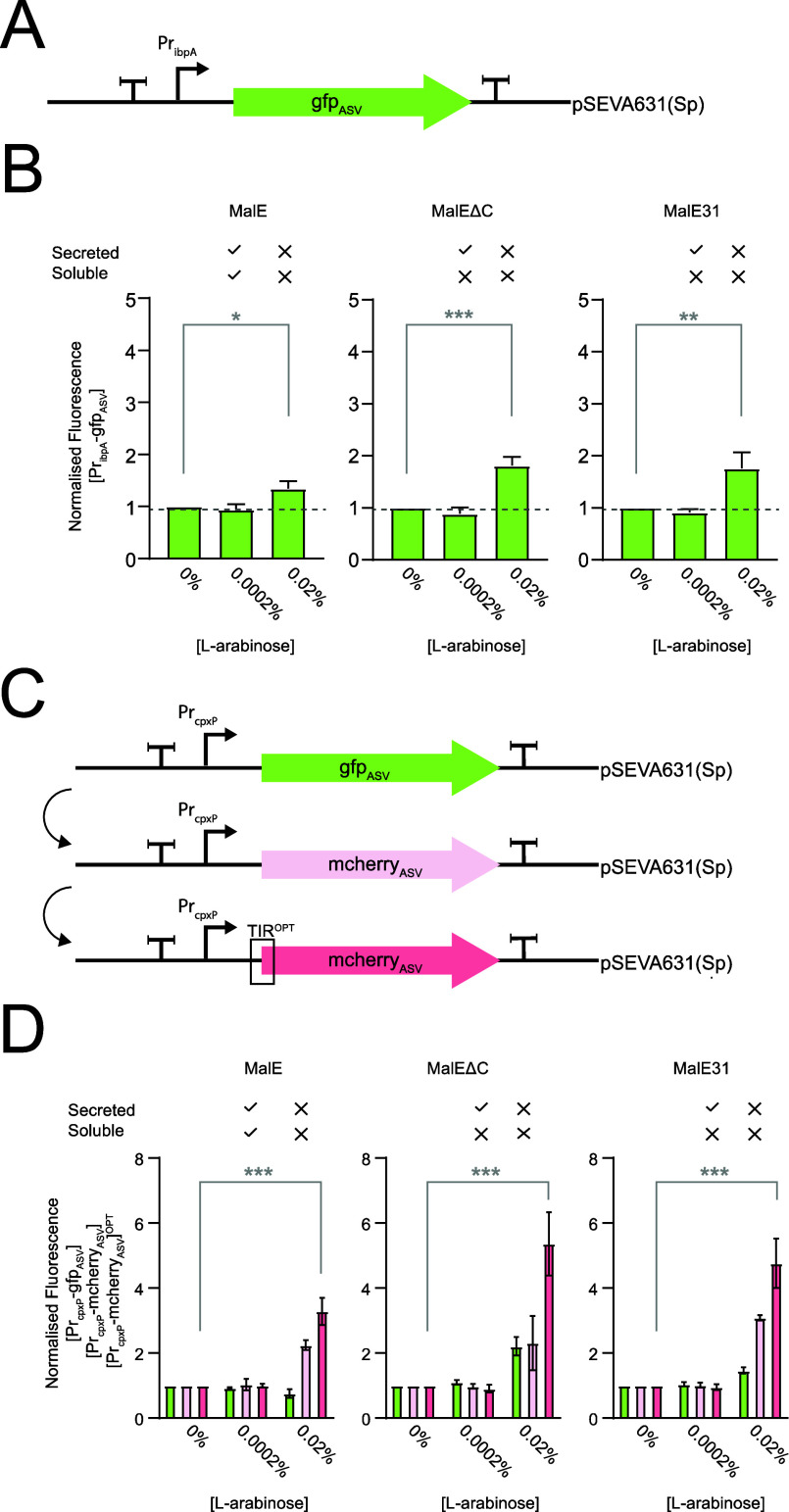
Genetic modules that can sense stress caused by inefficient secretion and periplasmic misfolding/aggregation. (A) Cartoon representation of the [Pr_ibpA_-gfp_ASV_] genetic module.^38^ The nucleotide sequence is available in the Supporting Information, Table S2. (B) Fluorescence readings from [Pr_ibpA_-gfp_ASV_] were taken when preMalE, preMalEΔC, and preMalE31 were induced for 3 h with 0% (no induction), 0.0002% (low induction), and 0.02% (w/v) (high induction) of l-arabinose. Fluorescence output per OD_600_ was normalized to the 0% sample (i.e., no induction). Ticks and crosses above the graphs indicate whether inefficient secretion or IB formation was observed by cell fractionations and Western blotting. Data presented as mean ± standard deviation (s.d.) (*n* ≥ 3). A statistically significant difference of *P* < 0.05 is denoted by *, *P* < 0.01 by **, and *P* < 0.001 by *** (two-tailed Student’s *t* test). No statistical difference is denoted n.s. (C) Cartoon representations of the [Pr_cpxP_-gfp_ASV_], [Pr_cpxP_-mcherry_ASV_], and [Pr_cpxP_-mcherry_ASV_]^OPT^ genetic modules. The nucleotide sequences are available in Supporting Information, Table S2. (D) Fluorescence readings from [Pr_cpxP_-gfp_ASV_], [Pr_cpxP_-mcherry_ASV_], and [Pr_cpxP_-mcherry_ASV_]^OPT^ were taken when preMalE, preMalEΔC, and preMalE31 were induced for 3 h with 0% (no induction), 0.0002% (low induction), and 0.02% (w/v) (high induction) l-arabinose. Fluorescence output per OD_600_ was normalized to the 0% (no induction) sample. Ticks and crosses above the graphs indicate whether inefficient secretion or IB formation was observed by cell fractionations and Western blotting. Data presented as mean ± standard deviation (s.d.) (*n* ≥ 3). A statistically significant difference of *P* < 0.05 is denoted by *, *P* < 0.01 by **, and *P* < 0.001 by *** (two-tailed Student’s *t* test). No statistical difference is denoted by n.s.

Our initial goal was to determine if we could identify a biosensor to monitor the inefficient secretion of preMalE, preMalEΔC, and preMalE31 from the cytoplasm. Previous work had noted that the heat shock response is activated when proteins with a signal peptide accumulate in the cytoplasm.^21^ We therefore used a genetic module that contained the heat shock-inducible promoter and 5′UTR for the inclusion body (IB)-binding protein IbpA fused to the coding sequence for an unstable version of the green fluorescent protein [P_ibpA_-gfp_ASV_]^38^ (Figure 2A). When preMalE, preMalE^ΔC^, and preMalE31 were induced with a low concentration of l-arabinose (0.0002%), the signal peptide was removed, indicating that they were efficiently secreted to the periplasm (Figure 1B) and the fluorescence signal from [P_ibpA_-gfp_ASV_] remained at background levels (Figure 2B). When preMalE, preMalEΔC, and preMalE31 were expressed with a high concentration of l-arabinose (0.02%), the signal peptide was partly retained, indicating that they were inefficiently secreted to the periplasm (Figure 1B) and the fluorescence from [P_ibpA_-gfp_ASV_] increased (Figure 2B). These data therefore indicate that [P_ibpA_-gfp_ASV_] can be used to monitor cellular stress caused when preMalE, preMalEΔC, and preMalE31 are inefficiently secreted from the cytoplasm.

The signal from [P_ibpA_-gfp_ASV_] was modest. We reasoned that this was because the 5′UTR for *ibpA* contains an RNA thermometer (the ROSE element) that represses translation initiation by sequestering the SD sequence.^39,40^ We attempted to amplify the signal from [P_ibpA_-gfp_ASV_] by varying the sequence around the ROSE element so that the SD was more accessible, but [P_ibpA_-gfp_ASV_] became unresponsive to the inefficient secretion of preMalE, preMalE^ΔC^, and preMalE^31^ (Supporting Information, Figure S1). All further experiments were therefore carried out with the original [P_ibpA_-gfp_ASV_].

### Stress Caused by Misfolding and Aggregation in the Periplasm can be Detected Using [P_cpxP_-mCherry_ASV_]^OPT^

Our next goal was to determine whether we could identify a biosensor that could monitor cellular stress caused by misfolding and aggregation of MalE, MalE^ΔC^, and MalE31 in the periplasm. Hunke and Betton had previously shown that the expression of preMalE31 elicited a Cpx stress response.^23^ We therefore engineered a [P_cpxP_-gfp_ASV_] genetic module and monitored fluorescence during the production of preMalE, preMalEΔC, and preMalE31 in the periplasm (Figure 2C). When preMalE was induced with a low concentration of l-arabinose (0.0002%), the signal peptide was removed, indicating that it was efficiently secreted to the periplasm (Figure 1B) and was soluble (Figure 1D). As anticipated, the fluorescence signal from [P_cpxP_-gfp_ASV_] remained at background levels (Figure 2D). When preMalEΔC and preMalE31 were induced with a low concentration of l-arabinose (0.0002%), the signal peptide was removed, indicating that they were efficiently secreted to the periplasm (Figure 1B) and that they were insoluble (Figure 1D). However, the fluorescence signal from [P_cpxP_-gfp_ASV_] still remained at background levels (Figure 2D). When preMalE, preMalEΔC, and preMalE31 were induced with a high concentration of l-arabinose (0.02%), they were partly secreted to the periplasm (Figure 1B), and the mature form was partly insoluble and partly soluble (Figure 1E). In these cells, the [P_cpxP_-gfp_ASV_] genetic sensor was activated (Figure 2D). This observation indicates that [P_cpxP_-gfp_ASV_] does not directly respond to misfolding and aggregation but is activated when misfolding and aggregation reach a threshold that is stressful to the cell.

*E. coli* possesses several other cell envelope stress responses (σ^E^, Rcs, Psp, and acid stress) which detect different physical, chemical, and biological stresses in the cell envelope and respond by reprogramming transcription to alleviate the stress.^41^ To determine if any of these stress responses were activated by protein misfolding and aggregation of MalE, MalE^ΔC^, and MalE31 in the periplasm, representative promoters were fused to the coding sequence of an unstable version of the green fluorescent protein as follows: [P_rpoE_-gfp_ASV_] (σ^E^), [P_rprA_-gfp_ASV_] (Rcs), [P_pspA_-gfp_ASV_] (Psp), and [P_hdeA_-gfp_ASV_] (acid stress).^41^ None of these sensors were activated when preMalE, preMalEΔC, and preMalE31 were induced with a high concentration of l-arabinose (0.02%), and they were not studied further (Supporting Information, Figure S2).

The [P_cpxP_-gfp_ASV_] genetic sensor was engineered to improve its performance. Initially, the coding sequence for GFP was replaced with that of mCherry (Figure 2C, middle). mCherry has a slower maturation time than GFPmut3, which we had used previously (*t*_1/2_ 15 vs 4.1 min; see https://www.fpbase.org and^42^). However, it fluoresces in the red spectrum and is therefore compatible with downstream applications using [P_ibpA_-gfp_ASV_] (see below). The output signal was then amplified by modifying the translation initiation region using a directed evolution approach (Figure 2C, bottom). The directed evolution approach identified a translation initiation region with increased efficiency, by randomizing the nucleotides around the AUG start codon for mCherry_ASV_ (Supporting Information, Figure S3).^43^ As a consequence, the translational output was increased from the same cognate promoter. The new genetic module was called [P_cpxP_-mcherry_ASV_]^OPT^. A high-level of expression from preMalE, preMalEΔC, and preMalE31 triggered a stronger response from [P_cpxP_-mcherry_ASV_]^OPT^ than it did from [P_cpxP_-mcherry_ASV_] and [P_cpxP_-gfp_ASV_] (Figure 2D).

### Biosensor that Detects Stress Caused by the Production of Proteins in the Periplasm

**Figure 3:**
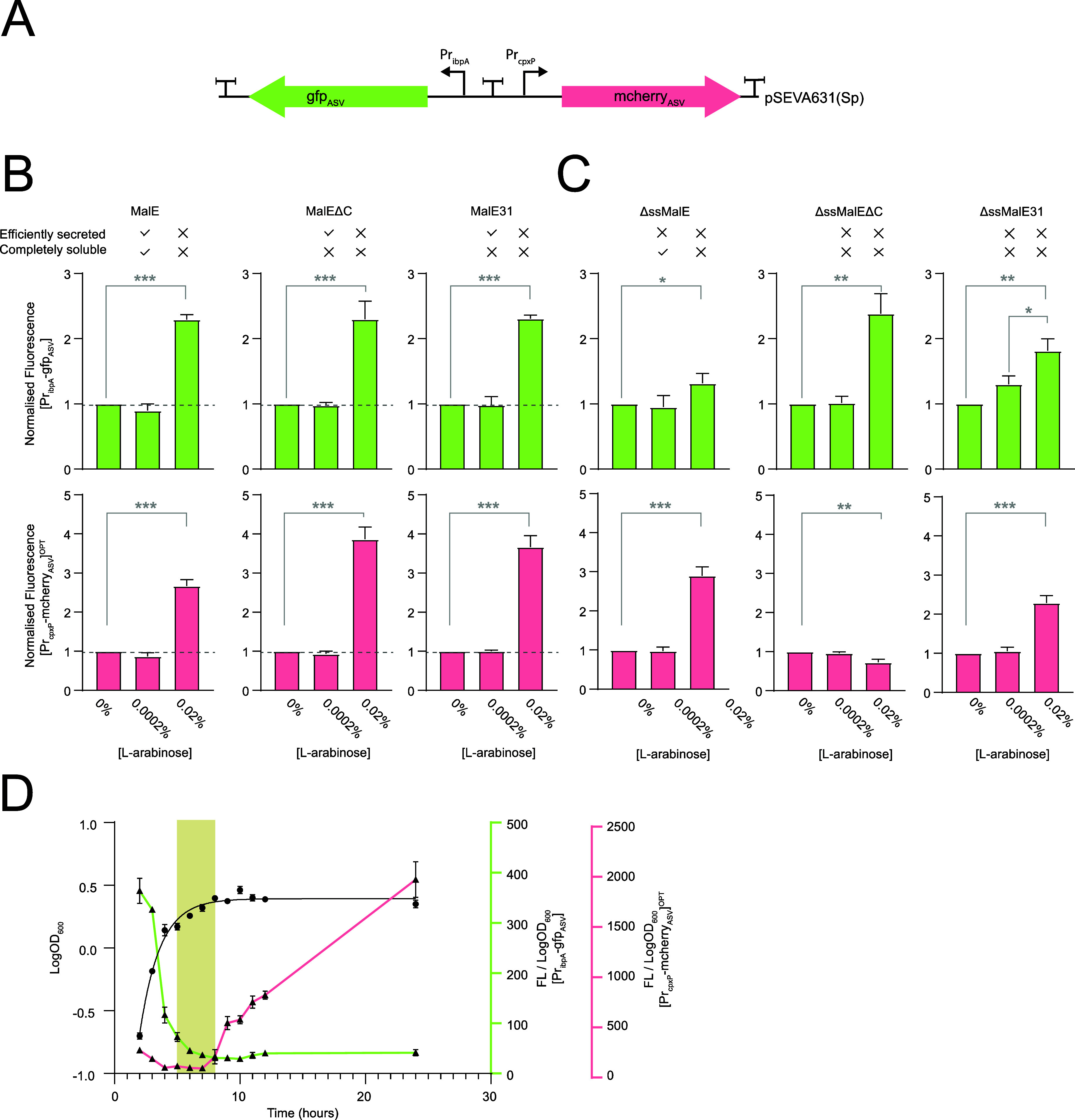
Construction and testing of pQC (plasmid for quality control) (A) Cartoon representation of the pQC biosensor. Transcriptional terminators used to isolate the genetic modules are indicated. The nucleotide sequence is available in Supporting Information, Table S2. (B) Fluorescence readings from pQC were taken when preMalE, preMalEΔC, and preMalE31 were induced for 3 h with 0% (no induction), 0.0002% (low induction), and 0.02% (w/v) (high induction) l-arabinose. The fluorescence output was normalized to the OD_600_ sample and then to the 0% (no induction) sample. Ticks and crosses above the graphs indicate whether the protein was efficiently secreted or totally soluble (i.e., no IBs were observed by cell fractionations and Western blotting). Data presented as mean ± standard deviation (s.d.) (*n* ≥ 3). A statistically significant difference of *P* < 0.05 is denoted by *, *P* < 0.01 by **, and *P* < 0.001 by *** (two-tailed Student’s *t* test). No statistical difference is denoted by n.s. (C) As for B, except that ΔssMalE, ΔssMalEΔC, and ΔssMalE31 were induced. (D) Background fluorescence from the [Pr_ibpA_-gfp_ASV_] and [Pr_cpxP_-mcherry_ASV_]^OPT^ genetic modules in pQC was measured during the growth of *E. coli*. The growth curve is marked in black. Fluorescence measured from [Pr_ibpA_-gfp_ASV_] is marked in green and that of [Pr_cpxP_-mcherry_ASV_]^OPT^ is marked in pink. Data presented as mean ± standard deviation (s.d.) (*n* ≥ 3). The beige stripe indicates that the window where pQC should be used as the background from both [Pr_ibpA_-gfp_ASV_] and [Pr_cpxP_-mcherry_ASV_]^OPT^ is low.

We engineered a biosensor plasmid that could monitor stress caused by multiple off-pathway events during the production of periplasmic proteins by cloning the [P_ibpA_-gfp_ASV_] and [P_cpxP_-mcherry_ASV_]^OPT^ genetic modules into the pSEVA631(Sp) backbone, separated by the secG leuU terminator^44^ (Figure 3A). The biosensor plasmid was called pQC (plasmid for quality control). Benchmarking of pQC indicated that the resulting fluorescence fingerprint was the same as that obtained when [P_ibpA_-gfp_ASV_] and [P_cpxP_-mcherry_ASV_]^OPT^ were tested individually (Figure 3B).

pQC could also be used to detect the aggregation of cytoplasmic proteins. When ΔssMalE, ΔssMalEΔC, and ΔssMalE31 were induced with a high concentration of l-arabinose, they generated an insoluble protein (Figure 1E), and the [P_ibpA_-gfp_ASV_] module was activated (Figure 3C). However, at a low concentration of l-arabinose, ΔssMalEΔC and ΔssMalE^31^ also generated insoluble proteins (Figure 1D), but the [P_ibpA_-gfp_ASV_] module was not activated (Figure 3C). The latter observation indicates that [P_ibpA_-gfp_ASV_] does not directly respond to misfolding and aggregation in the cytoplasm but is activated when misfolding and aggregation reach a threshold that is stressful to the cell. These observations are consistent with the work of Zutz and co-workers,^38^ and they underscore the dual use of [P_ibpA_-gfp_ASV_] as a genetic sensor for both cytoplasmic misfolding and cytoplasmic retention of proteins with a signal peptide (i.e., inefficient secretion). Notably the [P_cpxP_-mcherry_ASV_]^OPT^ module was also activated by ΔssMalE and ΔssMalE^31^ (but not ΔssMalEΔC) (Figure 1C). The molecular reason for these latter observations is not currently known. It could be that the heat shock (σ^32^) transcription factor recognizes the cpxP promoter (i.e., cross-talk) or that the Cpx stress response is activated by events in the cytoplasmic compartment (see refs (45 and 46)).

The fluorescence from pQC in the absence of recombinant protein production was also monitored at different stages of the growth cycle, so that we could better understand the background signal (Figure 3D). The [P_ibpA_-gfp_ASV_] genetic module gave a background signal in the exponential phase. In contrast, the [P_cpxP_-mCherry_ASV_]^OPT^ genetic module gave a background signal during the stationary phase (as described previously for P_cpxP_-^47^). At the late exponential phase, both [P_ibpA_-gfp_ASV_] and [P_cpxP_-mCherry_ASV_]^OPT^ gave a low background signal (Figure 3C). These observations indicate that measurements with pQC should ideally be made at the late exponential phase when the background is low.

### Optimizing the Production of Recombinant Proteins in the Periplasm Using pQC

**Figure 4:**
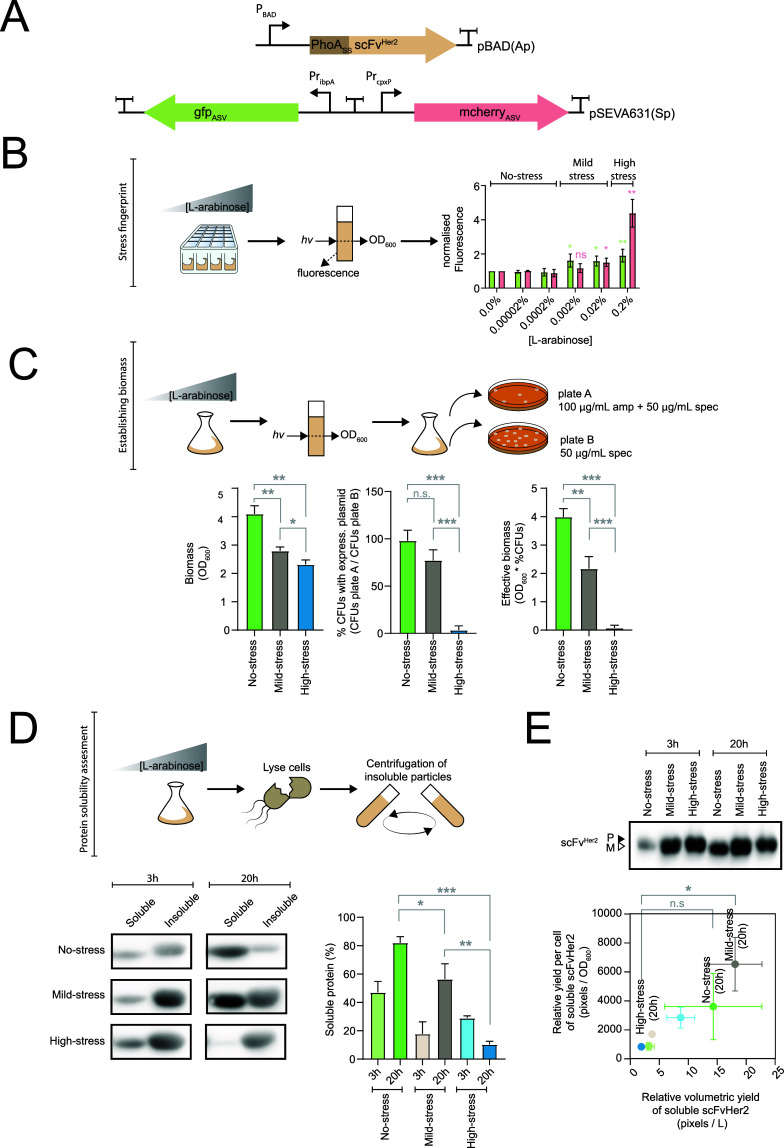
Optimizing the induction protocol for a single-chain antibody fragment that recognizes the human epidermal growth factor (scFvHer2) using pQC. (A) Cartoon representation of the plasmid used to express PhoA_ss_-scFv^Her2^ and the pQC biosensor used to monitor cellular stress. The nucleotide sequences are available in Supporting Information, Table S2. (B) Fluorescent “stress fingerprint" from pQC was captured when PhoA_ss_-scFv^Her2^ was expressed with varying concentrations of l-arabinose for 3 h. This allowed the identification of induction conditions that caused no stress, mild stress, and high stress to the cell (i.e., induction with 0.0002, 0.002, or 0.2% (w/v) l-arabinose, respectively). Data presented as mean ± standard deviation (s.d.) (*n* ≥ 3). A statistically significant difference to the uninduced control of *P* < 0.05 is denoted by *, *P* < 0.01 by **, and *P* < 0.001 by *** (two-tailed Student’s *t* test). No statistical difference is denoted by n.s. (C) Effect of stress on the accumulation of biomass was evaluated. Cells were induced for 20 h with a concentration of l-arabinose that caused no stress, mild stress, and high stress. The biomass was defined as the total number of OD_600_ units in the culture. The percentage of colony-forming units (cfus) in the culture that had retained both the pBAD expression plasmid and the pQC biosensor vs those that had only retained the pQC biosensor was determined as shown in the cartoon. Here, it was assumed that the viable but nonculturable (VBNC) phenotype did not influence the experiment. The effective biomass was defined as the biomass that had retained the pBAD expression plasmid and was capable of producing recombinant PhoA_ss_-scFv^Her2^. Data presented as mean ± standard deviation (s.d.) (*n* ≥ 3). A statistically significant difference of *P* < 0.05 is denoted by *, *P* < 0.01 by **, and *P* < 0.001 by *** (two-tailed Student’s *t* test). No statistical difference is denoted by n.s. (D) Solubility of recombinant scFv^Her2^ was assessed after 3 and 20 h in cells experiencing no stress, mild stress, and high stress. Top panel: cells were lysed and fractionated into soluble and insoluble fractions by centrifugation. Bottom left: an equal volume of the soluble and insoluble fractions was analyzed by SDS-PAGE and Western blotting with a HisProbe-HRP conjugate. Bottom right: the blots were quantified so that the % soluble protein could be determined [% soluble protein] = [# pixels soluble]/[# pixels soluble] + [# pixels insoluble]. Data presented as mean ± standard deviation (s.d.) (*n* ≥ 3). A statistically significant difference of *P* < 0.05 is denoted by *, *P* < 0.01 by **, and *P* < 0.001 by *** (two-tailed Student’s *t* test). No statistical difference is denoted by n.s. (E) Yield of scFv^Her2^ produced by cells experiencing no stress, mild stress, and high stress was determined. Top panel: a 50 μL aliquot of the culture expressing scFv^Her2^ was analyzed by SDS-PAGE and Western blotting with a HisProbe-HRP conjugate. The mature version (periplasmic localization) is denoted M, and the signal sequence-containing version (cytoplasmic localization) is denoted P. Bottom panel: the relative volumetric yield of soluble protein (*x*-axis) and the relative yield of soluble protein per cell were calculated from the blots and the ratio of soluble to insoluble protein [obtained in panel (E)]. Data presented as mean ± standard deviation (s.d.) (*n* ≥ 3). A statistically significant difference of *P* < 0.05 is denoted by *, *P* < 0.01 by **, and *P* < 0.001 by *** (two-tailed Student’s *t* test). No statistical difference is denoted by n.s. Data labels as in panel (D).

To demonstrate how pQC could be used to optimize the production of a periplasmic protein, we tested different induction conditions for a single-chain antibody fragment that recognizes the human epidermal growth factor (scFv^Her2^).^48^ In the experiment, the coding sequence for scFv^Her2^ was cloned into the pBAD expression plasmid downstream of the coding sequence of a PhoA signal peptide and then transformed into MC1061 cells containing pQC (Figure 4A). The cells were grown in 5 mL of LB media incubated in a 24-well plate at 37 °C with shaking. Cultures were initially induced for 3 h with increasing concentrations of l-arabinose, and the fluorescence fingerprint from pQC was captured (Figure 4B). At 0.0002% (w/v) l-arabinose (and lower concentrations), the fluorescence levels from both [P_ibpA_-gfp_ASV_] and [P_cpxP_-mCherry_ASV_]^OPT^ were comparable with those obtained from uninduced cells, indicating that the cells were not stressed by the production of scFv^Her2^ (Figure 4B). At 0.002% (w/v) l-arabinose (or higher concentrations), the fluorescence levels from pQC were higher than those obtained from uninduced cells, indicating that these cells were experiencing stress caused by both inefficient secretion and periplasmic aggregation during the production of scFv^Her2^. We arbitrarily classified the induction at 0.002% and 0.02% (w/v) l-arabinose as mild stress (one or both sensors were <3-fold above background), and the induction at 0.2% (w/v) l-arabinose as high-stress (one or both sensors was >3-fold above background).

The data above indicated that cells experienced stress from the inefficient secretion of scFv^Her2^ at 0.002% (w/v) l-arabinose and stress from periplasmic aggregation at 0.02% (w/v) l-arabinose. To gain more insight into this observation, we carried out a more extensive titration of scFv^Her2^, between 0.0002 and 0.002% (w/v) l-arabinose. This experiment indicated that the two stresses could not be consistently separated at 37 °C (Figure S4). However, when the induction was carried out at 20 °C, we observed that cells experienced stress from inefficient secretion prior to stress from protein aggregation in the periplasm (Figure S5). Thus, at 20 °C, the secretion capacity was exceeded before the folding capacity of the periplasm.

We also investigated how stress affected the accumulation of biomass during a longer production experiment. The cells were grown in 1 L of LB media incubated in a 2.5 L shaker flask at 37 °C with shaking and then induced with 0.0002, 0.002, or 0.2% (w/v) l-arabinose (i.e., inductions that caused no stress, mild stress, and high stress). We then measured the biomass and the percentage of cells that had maintained the pBAD expression plasmid (Figure 4C, top panel). After 20 h of induction, cells that were experiencing no stress produced the most biomass, followed by cells that were experiencing mild stress and then cells that were experiencing high stress (Figure 4C, left panel). The percentage of cells in the biomass that had maintained the pBAD expression plasmid was calculated by plating an aliquot of the cultures on LB agar with both ampicillin and spectinomycin or just spectinomycin and then comparing the colony numbers (Figure 4C, top panel). Approximately 98% of cells that were experiencing no stress maintained the pBAD expression plasmid after 20 h of induction, compared to approximately 77% for cells that were experiencing mild stress and <5% for cells that were experiencing high stress (Figure 4C, middle panel). Using these data, we calculated the effective biomass from 1 L of culture after 20 h of induction, which we define as the proportion of the biomass containing the pBAD expression plasmid and capable of producing recombinant scFv^Her2^. Cells that were experiencing no stress following the induction of scFv^Her2^ produced approximately two times more effective biomass than cells that were experiencing mild stress and approximately 50 times more biomass than cells that were experiencing high stress (Figure 4C, right panel).

The effective biomass under high-stress conditions could be increased by swapping the antibiotic resistance cassette of the pBAD expression plasmid from Tn3.12 (AmpR) to Tn903.1 (KanR) and selecting with kanamycin (Supporting Information, Figure S6). Kanamycin is still active in the culture media after >20 h, making it difficult for cells that have lost the expression plasmid to survive.^49^ In contrast, ampicillin (and other β-lactam antibiotics) is rapidly degraded, and cells that have lost the expression plasmid outgrow those that maintain it and are forced to produce recombinant proteins.^50,51^ However, using ampicillin is an advantage when cells are not stressed, as they do not become outgrown by plasmid-less cells and are able to generate >2 times more effective biomass than that when they are selected with kanamycin (Supporting Information, Figure S6).

We also asked how stress affected the quality of recombinant scFv^Her2^. The ratio of soluble to insoluble scFv^Her2^ was determined in cells that were experiencing no stress, mild stress, and high stress (Figure 4D). Quantification of the data indicated that, after either a 3 h or a 20 h induction, cells experiencing no stress produced the highest ratio of soluble scFv^Her2^. Analysis of the soluble protein by SDS-PAGE and Western blotting indicated that scFv^Her2^ obtained from non-stressed and highly stressed cells was indistinguishable during both oxidizing and reducing conditions. This observation most likely reflects the fact that disulfide bonds do not affect its migration in SDS-PAGE (Supporting Information, Figure S7). The localization of the soluble protein (periplasm vs cytoplasm) was difficult to discern by SDS-PAGE as PhoAss-scFv^Her2^ and scFv^Her2^ migrated similarly. However, fractionation confirmed that only the mature form (scFv^Her2^) was in the periplasm (PhoAss-scFvHer2 remained in the fraction with the spheroplasts and unbroken cells; Supporting Information, Figure S8).

The yield of soluble scFv^Her2^ in each experiment was also calculated (Figure 4E). After a 20 h induction, cells experiencing no stress and mild stress produced a higher volumetric yield as well as yield per OD_600_ than cells experiencing high stress. In contrast, after a 3 h induction, cells experiencing high stress produced higher volumetric yields of soluble scFv^Her2^ than cells experiencing no stress and mild stress.

Taken together, these data indicate that pQC could be used after 3 h of induction, when cells are in the late exponential phase of growth and the background signal is low, to identify induction conditions that resulted in an efficient and sustainable production process for scFv^Her2^. When a low concentration of l-arabinose was used for induction, the cells did not experience stress and were able to accumulate biomass, maintain the expression plasmid, and produce predominantly soluble scFv^Her2^. As the concentration of l-arabinose used for induction was increased, the cells started to experience stress. Cells experiencing mild stress were able to produce comparable titers of soluble scFv^Her2^ as those experiencing no stress; however, they were less efficient at producing biomass and maintaining the expression plasmid. Cells experiencing high-stress were unable to produce comparable amounts of soluble scFv^Her2^ and were even less efficient at producing biomass and maintaining the expression plasmid.

**Figure 5:**
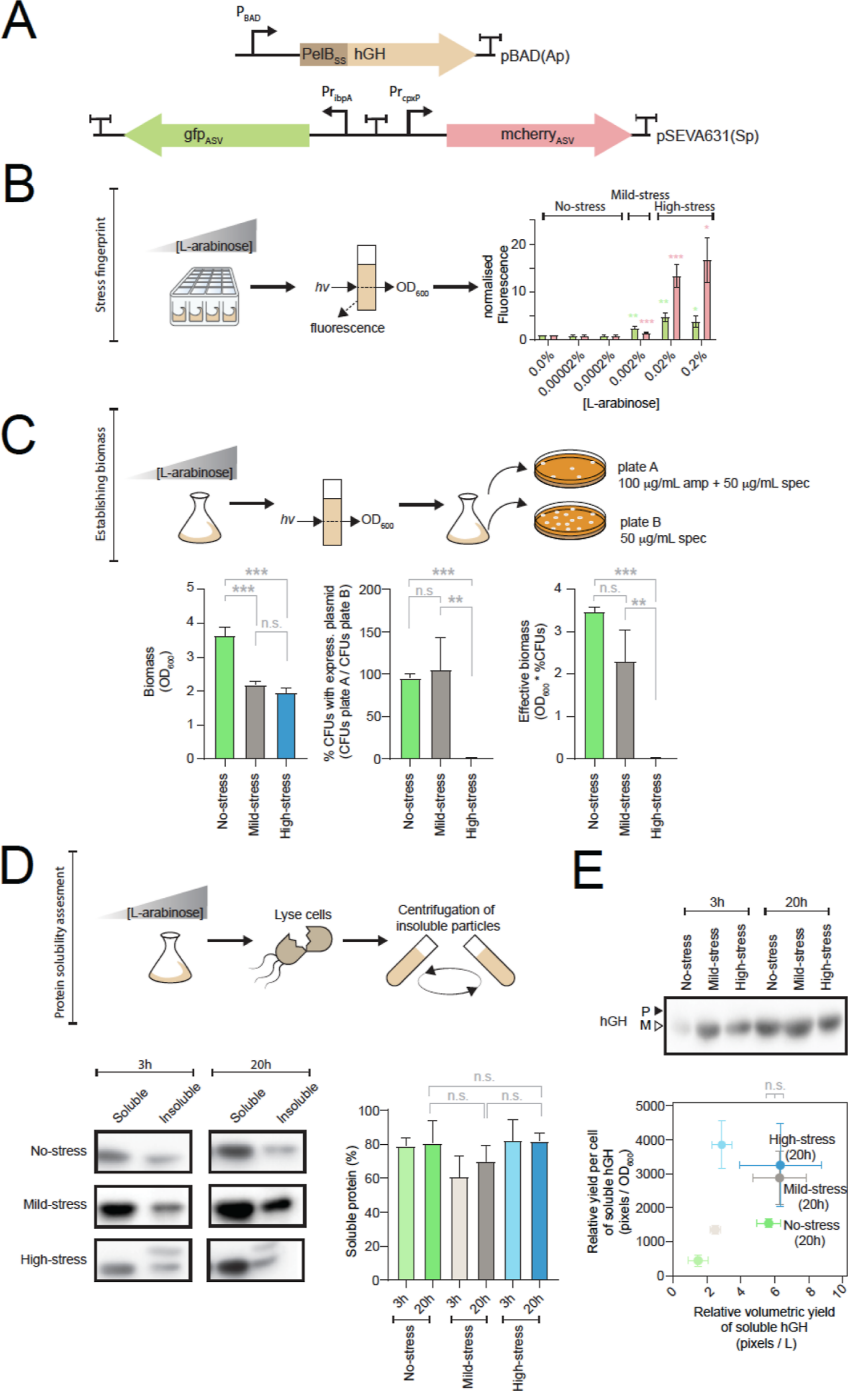
Optimizing the induction protocol for hGH using pQC. (A) Cartoon representation of the plasmid used to express PelB_ss_-hGH and the pQC biosensor used to monitor cellular stress. The nucleotide sequences are available in Supporting Information, Table S2. (B) Fluorescent “stress fingerprint” from pQC was captured when PelB_ss_-hGH was expressed with varying concentrations of l-arabinose for 3 h. This allowed the identification of induction conditions that caused no stress, mild stress, and high stress to the cell (i.e., induction with 0.0002, 0.002, or ≥0.02% (w/v) l-arabinose, respectively). Data presented as mean ± standard deviation (s.d.) (*n* ≥ 3). A statistically significant difference to the uninduced control of *P* < 0.05 is denoted by *, *P* < 0.01 by **, and *P* < 0.001 by *** (two-tailed Student’s *t* test). No statistical difference is denoted by n.s. (C) Effect of stress on the accumulation of biomass was evaluated. Cells were induced for 20 h with a concentration of l-arabinose that caused no stress, mild stress, and high stress. The biomass was defined as the total number of OD_600_ units in the culture. The percentage of cfus in the culture that had retained both the pBAD expression plasmid and the pQC biosensor vs those that had only retained the pQC biosensor was determined as shown in the cartoon. Here, it was assumed that the VBNC phenotype did not influence the experiment. The effective biomass was defined as the biomass that had retained the pBAD expression plasmid and was capable of producing recombinant PelB_ss_-hGH. Data are presented as mean ± standard deviation (s.d.) (*n* ≥ 3). A statistically significant difference of *P* < 0.05 is denoted by *, *P* < 0.01 by **, and *P* < 0.001 by *** (two-tailed Student’s *t* test). No statistical difference is denoted by n.s. (D) Solubility of recombinant hGH was assessed after 3 and 20 h in cells experiencing no stress, mild stress, and high stress. Top panel: cells were lysed and fractionated into soluble and insoluble fractions by centrifugation. Bottom left: an equal volume of the soluble and insoluble fractions was analyzed by SDS-PAGE and Western blotting with a HisProbe-HRP conjugate. Bottom right: the blots were quantified so that the % soluble protein could be determined. [% soluble protein] = [# pixels soluble]/[# pixels soluble] + [# pixels insoluble]. Data presented as mean ± standard deviation (s.d.) (*n* ≥ 3). A statistically significant difference of *P* < 0.05 is denoted by *, *P* < 0.01 by **, and *P* < 0.001 by *** (two-tailed Student’s *t* test). No statistical difference is denoted by n.s. (E) Yield of hGH produced by cells experiencing no stress, mild stress, and high stress was determined. Top panel: a 50 μL aliquot of the culture expressing hGH was analyzed by SDS-PAGE and Western blotting with a HisProbe-HRP conjugate. The mature version (periplasmic localization) is denoted M, and the signal sequence-containing version (cytoplasmic localization) is denoted P. Bottom panel: the relative volumetric yield of soluble protein (*x*-axis) and the relative yield of soluble protein per cell were calculated from the blots and the ratio of soluble to insoluble protein [obtained in panel (E)]. Data presented as mean ± standard deviation (s.d.) (*n* ≥ 3). A statistically significant difference of *P* < 0.05 is denoted by *, *P* < 0.01 by **, and *P* < 0.001 by *** (two-tailed Student’s *t* test). No statistical difference is denoted by n.s. Data labels as in panel (D).

We also used pQC to optimize the induction conditions for the human growth hormone (hGH). In this experiment, the coding sequence for hGH was cloned into the pBAD expression plasmid downstream of the coding sequence of a PelB signal peptide (Figure 5A). The cells were grown in 5 mL of LB media incubated in a 24-well plate at 37 °C with shaking. Cultures were again induced for 3 h with increasing concentrations of l-arabinose, and the stress fingerprint from pQC was obtained (Figure 5B). At 0.0002% (w/v) l-arabinose (and lower concentrations), the fluorescence levels from both [P_ibpA_-gfp_ASV_] and [P_cpxP_-mCherry_ASV_]^OPT^ were comparable with those obtained from uninduced cells, indicating that the cells were not stressed by the production of hGH. At 0.002% (w/v) l-arabinose (or higher concentrations), the fluorescence levels from pQC were higher than those obtained from uninduced cells, indicating that these cells were experiencing various degrees of cellular stress during the production of hGH. We arbitrarily classified the induction at 0.002% (w/v) l-arabinose conditions as mild stress (one or both sensors were <3-fold above background) and the induction at 0.02% and 0.2% (w/v) l-arabinose as high stress (one or both sensors were >3-fold above background).

The data above indicated that cells experienced stress from both the inefficient secretion of hGH and periplasmic aggregation at 0.02% (w/v) l-arabinose. To gain more insight into this observation, we carried out a more extensive titration of hGH, between 0.0002 and 0.002% (w/v) l-arabinose. This experiment indicated that the two stresses could not be easily separated at 37 °C (Figure S9). However, when the induction was carried out at 20 °C, we again observed that cells experienced stress from inefficient secretion, prior to stress from protein aggregation in the periplasm (Figure S10). Thus, at 20 °C, the secretion capacity was again exceeded before the folding capacity of the periplasm.

As we had done previously, we analyzed how stress affected the accumulation of effective biomass during a production experiment. The cells were again grown in 1 L of LB media incubated in a 2.5 L shaker flask at 37 °C with shaking. After 20 h of induction, cells that were experiencing no stress following the induction of hGH produced approximately 1.5 times more effective biomass than cells that were experiencing mild stress and approximately 200 times more effective biomass than cells that were experiencing high stress (Figure 5C, right panel). Experiments showing effective biomass when kanamycin was used to select the pBAD expression plasmid are available in the Supporting Information (Figure S11). Cell fractionation experiments indicated that cells experiencing no stress, mild stress, and high stress produced predominantly soluble protein (Figure 5D). This soluble protein was judged to be in the periplasm as it did not contain a signal peptide, and fractionation confirmed that only the mature form (hGH) was in the periplasm (PelB-hGH remained in the fraction with the spheroplasts and unbroken cells; Supporting Information, Figure S8). Moreover, the soluble hGH obtained from nonstressed and highly stressed cells gave a characteristic gel shift under oxidizing and reducing conditions, indicating that it contained disulfide bonds (Supporting Information, Figure S12). Finally, there was no difference in the volumetric yield of soluble hGH between cells experiencing no stress, mild stress, and high stress (Figure 5E).

Taken together, these data indicate that pQC could be used (in the late exponential phase of growth) to identify induction conditions that resulted in an efficient and sustainable (i.e., long running) production process for hGH. When a low concentration of l-arabinose was used for induction, the cells did not experience stress and were able to accumulate biomass, maintain the expression plasmid, and produce predominantly soluble hGH. As the concentration of l-arabinose used for induction was increased, the cells started to experience stress. Cells experiencing mild stress and high stress were able to produce comparable titers of soluble hGH as cells experiencing no stress but were less efficient at maintaining the expression plasmid.

## Discussion

The production of recombinant periplasmic proteins is a challenging endeavor. In many experiments, inefficient secretion from the cytoplasm and/or misfolding in the periplasm are common off-pathway outcomes.^4,20^ When these occur, the cell activates stress responses that initiate transcriptional reprogramming.^21,23^ Stress-induced transcriptional reprogramming is in place to help the cell adapt and ultimately regain homeostasis. However, since the genetic modules used to produce recombinant periplasmic proteins are not hard-wired in the genome, they are not regulated by stress-induced transcriptional reprogramming. Off-pathway outcomes therefore cause persistent stress that affects cellular energy metabolism and cell growth.^22^

A simple strategy to avoid stress and its detrimental effects is to tune the expression levels of the recombinant periplasmic protein to the capacity of the cell.^19,24^ This requires that relevant stresses be monitored during production. Herein, we have engineered a biosensor from (1) the [P_ibpA_-gfp_ASV_] genetic module,^38^ which monitors the heat shock response (σ^32^) and is activated when proteins are inefficiently secreted from the cytoplasm, and (2) the [P_cpxP_-mcherry_ASV_]^OPT^ genetic module (described here), which monitors the Cpx extra-cytoplasmic stress response and is activated when proteins misfold/aggregate in the periplasm. In the study, the biosensor was inserted into a plasmid called pQC (plasmid for quality control) and evaluated by titrating the expression levels of preMalE, preMalE^ΔC^, preMalE^31^, PhoAss-scFv^Her2^, and PelBss-hGH. By monitoring the dual-color fluorescent fingerprint, we were able to identify induction conditions that did not activate [P_ibpA_-gfp_ASV_] and [P_cpxP_-mcherry_ASV_]^OPT^, as well as those that did. In our titrations, we observed that the activation of both [P_ibpA_-gfp_ASV_] and [P_cpxP_-mcherry_ASV_]^OPT^ occurred at the same induction concentration when cells were grown at 37 °C, indicating that the secretion capacity of the Sec translocon and the periplasmic folding capacity of the cell were being exceeded simultaneously. However, at 20 °C, we observed that the secretion capacity was limiting, but the periplasmic folding capacity of the cell had not been reached. These capacities could be further pushed by manipulating the media composition or using protein engineering strategies (i.e., signal peptides, solubility tags, and coexpression of chaperones).^8,15,16^ Although we have not explored these strategies here, we believe that pQC would be useful as a screening tool when evaluating them.

It has previously been demonstrated that avoiding stress during the production of proteins in both the cytoplasm and periplasm results in more biomass.^22,52,53^ In this study, we also noted that avoiding stress during the production of PhoAss-scFv^Her2^ and PelBss-hGH resulted in more effective biomass, the biomass that maintained the expression plasmid and was (in principle) capable of producing recombinant protein. Over 20 h induction, cells experiencing no stress produced approximately 1.5–2-fold more effective biomass than cells experiencing mild stress and 50–200-fold more effective biomass than cells experiencing high stress. The effective biomass in stressed cells could be increased by using an expression plasmid containing the Tn903.1 cassette (KanR) and selecting with kanamycin (we initially used the Tn3.12 cassette (AmpR) and selected with ampicillin). Kanamycin lasts longer in the culture than β-lactam antibiotics, and expression plasmids containing the Tn903.1 cassette are retained with a higher efficiency than those containing the Tn3-type cassettes.^49,51^ There are potentially other advantages for cells experiencing no stress. For example, those cells that have maintained the expression plasmid will be less likely to propagate mutations that downregulate expression levels or kill the cell.^54^ For PhoAss-scFv^Her2^, we also observed that, over a 20 h induction, cells experiencing no stress (i.e., when the fluorescence fingerprint from pQC was measured), produced a higher proportion of soluble protein than cells experiencing mild stress and high stress. Given these data, we believe that pQC will be broadly useful for identifying sustainable production conditions for recombinant periplasmic proteins.

Promoter regions derived from nature (such as P_ibpA_- and P_cpxP_-) have notoriously subtle transcriptional responses, as this is what is required in the context of the cell. For bioengineering and industrial applications, a stronger and more robust output is usually desired. In this study, the output signal from [P_cpxP_-mcherry_ASV_] was amplified to improve the signal-to-noise ratio. This was done using a directed evolution approach to optimize the efficiency of the TIR in [P_cpxP_-mcherry_ASV_] which increased the efficiency of translation without affecting the transcriptional response. We observed that the output signal from [P_cpxP_-mcherry_ASV_]^OPT^ was two- to threefold higher than that from [P_cpxP_-mcherry_ASV_], from the same input. This methodology (see refs (43,55)) has also been used previously to amplify the output signal from the T7 and pBAD promoters.^56,57^ We anticipate that it will be useful for other transcription-based biosensors.

The study noted three limitations with the [P_ibpA_-gfp_ASV_]/[P_cpxP_-mcherry_ASV_]^OPT^ biosensor, which potential users should be aware of.

1. [P_ibpA_-gfp_ASV_] and [P_cpxP_-mcherry_ASV_]^OPT^ do not directly respond to inefficient secretion and protein misfolding in the periplasm but rather the stresses caused by them. Thus, there will be a threshold for the activation of these genetic molecules.
2. The background from both the [P_ibpA_-gfp_ASV_] and [P_cpxP_-mcherry_ASV_]^OPT^ genetic modules changes during the growth cycle (i.e., in uninduced cells). For [P_ibpA_-gfp_ASV_], we observed background signal during early exponential growth when cells are growing fast. To our knowledge, this observation has not been made previously, but it is not surprising since cells produce native proteins and misfolding can occur. For comparison, slow growing cells (i.e., in the lag phase) do not produce native proteins to the same extent and they do not exhibit background transcription from the heat shock response.^58^ For [P_cpxP_-mCherry_ASV_]^OPT^, we observed background signal during the stationary phase. This observation is consistent with previous work,^47^ but the molecular reason why the Cpx stress response is activated in the stationary phase of growth is not yet clear.^46^ To facilitate the interpretation of the fluorescent fingerprint from pQC, we carried out measurements at a time point when the background from both modules was low, and we subtracted these values.
3. It is not yet clear if [P_ibpA_-gfp_ASV_] and [P_cpxP_-mcherry_ASV_]^OPT^ can be used to monitor the production of all periplasmic proteins. In this study, we demonstrated that off-pathway events could be detected during the production of preMalE, preMalE^ΔC^, preMalE^31^, PhoAss-scFv^Her2^, and PelB-hGH. We speculate that the biosensor will work for most other recombinant periplasmic proteins; however, this remains to be determined.

Finally, it should be noted that the biosensor in pQC may have alternative uses not fully described here. [P_ibpA_-gfp_ASV_] is activated by the heat shock response and can also be used for monitoring the misfolding and aggregation of cytoplasmic proteins.^38^ [P_cpxP_-mcherry_ASV_]^OPT^ is activated by the Cpx envelope stress response and can be used for monitoring the misfolding of membrane proteins.^45,59^ This genetic module can also be useful for studying bacterial physiology, such as the function of periplasmic chaperones (see ref (60)) or the effect of lipid perturbations (see ref (61)).

## Methods

### Molecular Cloning

All polymerase chain reactions (PCR) were performed using the Q5 high-fidelity DNA polymerase (New England Biolabs). All DNA oligonucleotide primers were synthesized by Eurofins Genomics (Germany). A list of primers used in this study can be found in the Supporting Information, Table S1. All DNA sequencing was carried out by Eurofins Genomics (Germany).

The expression plasmid for producing preMalE was generated by PCR amplification of the pBAD backbone using the P1 and P2 primers and PCR amplification of the *malE* coding sequence from the *E. coli* strain MG1655 using the P3 and P4 primers and then assembling them using the *in vivo* cloning technique.^62^ The expression plasmids for producing preMalE^31^ were generated by including a mismatch of four base pairs (GAAT to ATCC) in the expression plasmids for producing preMalE using the P5 and P6 primers. This resulted in two amino acid substitutions: G58 to D58 and I59 to P59. The construct preMalE^ΔC^ was generated by PCR using primers P7 and P8. A subsequent cloning mistake was fixed by removing a single base using primers P9 and P10. The signal sequences were subsequently removed using primers P11 and P12 to generate plasmids that produced ΔssMalE, ΔssMalE^31^, and ΔssMalE^ΔC^. In all of these expression plasmids, the Tn3.12 cassette, which contains the coding sequence for β-lactamase, was converted to a MINimally expressed Tn3.12^MIN^ version by mismatch PCR using the P13 and P14 primers (as described in ref (63)). As a result, these plasmids were maintained by using 20 μg/mL ampicillin.

The pSEVA631(Sp) [P_ibpA_-gfp_ASV_] genetic sensor plasmid^38^ was a gift from Alex Toftgaard Nielsen (Danish Technical University). The genetic sensor library presented in Supporting Information, Figure S1 was created by replacing the promoter region for ibpA (P_ibpA_-) with another promoter region. This was done by PCR amplification of the plasmid backbone using the P15 and P16 primers, and PCR amplification of the promoter sequence from the genome of BL21(*DE3*) using primers from P17–P26, and then ligating the fragments using the *in vivo* cloning technique.^62^ Promoter regions were chosen as they represented a known stress response and were defined as the nucleotides annotated as promoters or TF binding sites in RegulonDB.^64^ These plasmids were maintained using 50 μg/mL of spectinomycin.

The pSEVA631(Sp) [P_cpxP_-mcherry_ASV_] plasmid was created by removing the coding sequence for GFP from pSEVA631(Sp) [P_cpxP_-gfp_ASV_] and replacing it with the coding sequence of mCherry. This was done by PCR amplification of the plasmid backbone using the P27 and P28 primers, and PCR amplification of the coding sequence for mCherry from pET28-mCherry^56^ using primers P29 and P30, and then ligating the fragments using the *in vivo* cloning technique.^62^

pQC was created in three steps. First, [P_cpxP_-mcherry_ASV_] was amplified by PCR from pSEVA631(Sp) [P_cpxP_-mcherry_ASV_] using the P31 and P32 primers. pSEVA631(Sp) [P_ibpA_-gfp_ASV_] was amplified by PCR using the P33 and P34 primers. The two fragments were ligated (in a back-to back orientation) using the *in vivo* cloning technique.^62^ In a second step, the secG terminator was amplified by PCR from the genome of BL21(*DE3*) using primers P35 and P36. The backbone of pSEVA631(Sp) [P_ibpA_-gfp_ASV_] [P_cpxP_-mcherry_ASV_] was amplified by PCR using P37 and P38 primers. The two fragments were ligated using the *in vivo* cloning technique.^62^ Finally, the TIR spectrum in [P_cpxP_-mcherry_ASV_] was changed to the D4 variant by mismatch PCR using primers P39 and P40.

The expression plasmids for producing PelBss-hGH and PhoAss-scFv^Her2^ were described previously.^12^ All coding sequences used in the study are available in the Supporting Information, Table S2.

### Synthetic Evolution of the TIR

Changes in the TIR region of pSEVA631(Sp) [P_cpxP_-mcherry_ASV_] were introduced by PCR with degenerate primers. The primers P40 and P41 were used to completely randomize six nucleotides upstream of the ATG start codon and synonymously randomized two codons downstream of the ATG. The PCR was performed with 30 cycles of 95 °C: 30 s, 45 °C: 30 s, and 72 °C: 300 s. Twenty-five microliters of the PCR product was treated with 20 units of Dpn1 for 90 min before transformation into chemically competent MC1061 cells harboring pBAD-malE^31^. 190 colonies of the transformants were picked and used to inoculate 500 μL of LB broth containing 50 μg/mL spectinomycin and 20 μg/mL ampicillin in 2.2 mL 96-well plates. These cultures were then grown overnight at 37 °C with shaking at 185 rpm. The overnight cultures were back-diluted 1:100 in 5 mL of LB broth containing antibiotics in 24-well plates. The cultures were grown at 37 °C with shaking at 185 rpm to an OD_600_ between 0.3 and 0.7. Each culture was then induced with 0.02% (w/v) l-arabinose for 3 h at 37 °C with shaking at 185 rpm. The OD_600_ and fluorescence were measured (as described below) and compared to data obtained using the original pSEVA631(Sp) [P_cpxP_-mcherry_ASV_] and pBAD-malE^31^.

The plasmids from the top performing 15 clones were purified and used to retransform chemically competent MC1061 cells. Three colonies from each transformation (biological replicates) were used to inoculate 500 μL of LB broth containing antibiotics. These cultures were then grown overnight at 37 °C with shaking at 185 rpm. Overnight cultures were back-diluted 1:100 to start two sets of 5 mL cultures of LB broth containing antibiotics in 24-well plates. The cultures were grown at 37 °C with shaking at 185 rpm to an OD_600_ between 0.3 and 0.7. One set was then induced with 0.02% (w/v) l-arabinose, and the other set remained without l-arabinose. These cultures were grown for a further 3 h at 37 °C with shaking at 185 rpm before OD_600_, and fluorescence were measured. To isolate the selected pSEVA631(Sp) [P_cpxP_-mcherry_ASV_]^OPT^ plasmid from the pBAD-malE^31^ plasmid, cells were plated on LB agar containing only 50 μg/mL spectinomycin, and the plasmid was isolated and sequenced.

### Protein Expression and Cell Fractionation

Coding sequences cloned into pBAD expression plasmids were transformed into the MC1061 strain (str.K-12F^–^ λ^–^ Δ*(ara-leu)7697* [*araD139*]B/rΔ*(codB-lacI)3 galK16 galE15* e14^–^*mcrA0 relA1 rpsL150*(Str^R^) *spoT1 mcrB1 hsdR2*(*r*^–^*m*^+^)), either alone or together with a pSEVA631(Sp)-based genetic sensor plasmid, using a standard heat shock protocol.

For screening, a single colony was used to inoculate 500 μL of LB (5 g/L yeast extract, 10 g/L tryptone, 10 g/L NaCl) containing either 20 μg/mL ampicillin (MalE and variants) or 100 μg/mL ampicillin (PelBss-hGH and PhoAss-scFv^Her2^) in 2.2 mL 96-well plates. When the pSEVA631(Sp)-based genetic sensor plasmid was present, 50 μg/mL spectinomycin was also added. Cultures were grown at 37 °C while being shaken at 185 rpm for 16–20 h. A 50 μL aliquot was back-diluted into 5 mL of fresh LB containing appropriate antibiotics in a 5 mL 24-well plate. Cultures were grown at 37 °C with shaking at 185 rpm to an OD_600_ between 0.3 and 0.7 before induction with varying concentrations of l-arabinose for 3 h. The OD_600_ and fluorescence were measured as described below.

For cell fractionations of MalE and variants, cultures were scaled up to 25 mL in 250 mL Erlenmeyer flasks. Cell pellets were harvested at 3220*g* for 10 min at 4 °C. Pellets were resuspended in 900 μL of Tris-buffer (50 mM Tris, pH 8.3, 100 mM NaCl) and 100 μL of lysozyme from chicken egg white (Sigma-Aldrich), followed by 40 min incubation at 4 °C. Resuspended cells were lysed by sonication using an ultrasonic processor VCX130 (Sonics & Materials, Inc.) at 10 s on/off for 5 min with 70% intensity. Sonicated samples were centrifuged at 17,000*g* for 3 min at 4 °C to remove unbroken cells. A 150 μL aliquot of the supernatant was saved as the total fraction. A 600 μL aliquot of the remaining supernatant was transferred to a Beckman ultracentrifuge tube and centrifuged at 22,000*g* for 1 h at 4 °C (Optima MAX-XP with TLA55 rotor, Beckman coulter), and 150 μL aliquot of the supernatant was saved as the soluble fraction. The pellet was resuspended in 600 μL of Tris-buffer, and a 150 μL aliquot was saved as the insoluble fraction.

For fractionation of cells of PelBss-hGH and PhoAss-scFv^Her2^, cultures were scaled up to 1 L in 2.5 L shaker flasks. Cell pellets from 500 mL of culture were collected by centrifugation at 4000*g* for 20 min at 4 °C and resuspended in 50 mL of Tris-buffer (50 mM Tris, pH 8.3, 100 mM NaCl). Fifty units of DNase1 were added, and the cell suspension was made homogeneous using a glass Dounce homogenizer. Cells were lysed by three passes through an Avestin emulsiflex C3 high-pressure homogenizer (Avestin, Canada) operating at 10,000–15,000 PSI. Unlysed cells were separated by centrifugation at 3220*g* for 10 min at 4 °C, and the pellet was discarded. The total lysate was collected by aspiring a 150 μL aliquot of the lysate. A 35 mL aliquot of the remaining lysate was separated into soluble and insoluble fractions by centrifugation at 22,000*g* for 1 h at 4 °C. An aliquot of the soluble sample was collected by aspiring a 150 μL aliquot of the supernatant. The pellet was resuspended in 35 mL of Tris-buffer (50 mM Tris, pH 8.3, 100 mM NaCl), and a 150 μL aliquot of the insoluble sample was collected.

Periplasmic fractions were prepared using a protocol described in ref (65). A 50 μL aliquot of culture was back-diluted into 5 mL of fresh LB containing appropriate antibiotics in a 5 mL 24-well plate. Cultures were grown at 37 °C with shaking at 185 rpm to an OD_600_ of approximately 0.5 and then induced with l-arabinose for 3 h (preMalE) or 20 h (PhoAss-scFv^Her2^, PelBss-hGH). A volume equivalent to an OD_600_ of 2 was collected by centrifugation at 8000*g* for 20 min at 4 °C and resuspended in 24 μL of TSE buffer [200 mM Tris–HCl pH 8, 500 mM sucrose, 1 mM ethylenediaminetetraacetic acid (EDTA)] and incubated at room temperature for 10 min. Cold shock was then carried out by adding 24 μL of ice-cold water and incubating the samples on ice for 10 min. The supernatant containing the periplasmic fraction was separated from the spheroplasts and unbroken cells by centrifugation at 8000*g* for 20 min at 4 °C. Samples were suspended in Laemmli buffer, boiled, and then separated by SDS-PAGE.

### Quantification of Fluorescence from the Genetic Sensors

A 1 mL aliquot of bacterial culture was collected by centrifugation at 3220*g* for 15 min. The culture medium was removed, and the pelleted cells were resuspended in 200 μL of buffer (50 mM Tris–HCl pH 8.0, 200 mM NaCl, 15 mM EDTA). The cell suspension was transferred to a 96-well optical bottom black-wall plate (Thermo Scientific), and the fluorescence was determined by a SpectraMax Gemini EM plate reader (Molecular Devices, U.K.). Fluorescence from [P_ibpA_-gfp_ASV_] was measured with an excitation wavelength of 475 nm and emission wavelength of 515 nm. A long-pass emission cutoff filter of 495 nm was used to reduce background. Fluorescence from [P_cpxP_-mcherry_ASV_] was measured with an excitation wavelength of 585 nm and an emission wavelength of 610 nm. A long-pass emission cutoff filter of 595 nm was used to reduce background. Fluorescence values were normalized by the optical density of the cultures (OD_600_). These values were obtained from a 200 μL aliquot of bacterial culture using a SpectraMax *m2e* plate reader (Molecular Devices, U.K.) at 600 nm. Finally, the fluorescence per OD_600_ was normalized to the control sample (i.e., cells not expressing any recombinant protein).

### Plasmid Maintenance

To assess the proportion of cells in the culture that contained the pBAD expression plasmid, a single colony of MC1061 containing pBAD-PelBss-hGH/pQC or pBAD-PhoAss-scFv^Her2^/pQC was grown in 10 mL of LB media supplemented with 50 μg/mL spectinomycin and either 100 μg/mL ampicillin or 50 μg/mL kanamycin for 16–20 h at 37 °C with agitation at 185 rpm in a 50 mL tube. The cultures were back-diluted 1:100 in 1 L of LB media supplemented with antibiotics and grown to an OD_600_ of approximately 0.5. The cells were then induced with the appropriate concentration of l-arabinose (see text for details) and cultured for further 20 h. An aliquot of the culture was subsequently plated out on LB agar with 50 μg/mL of spectinomycin and separately on another plate, with either 50 μg/mL spectinomycin and 100 μg/mL ampicillin or 50 μg/mL spectinomycin and 50 μg/mL kanamycin. Images were taken using the upper white light in a GenoPlex (VWR International), and the number of colonies was counted using the Fiji software.^66^ Plasmid maintenance = [# colonies on plate with 50 μg/mL spectinomycin and 100 μg/mL ampicillin/# colonies on plate with 50 μg/mL spectinomycin] ×100.

### SDS PAGE and Western Blotting

SDS-PAGE was performed using 1 mm 12% Tris-glycine acrylamide gels run on an Hoefer Mighty Small II Mini Vertical Electrophoresis System, and all gels were run at 100 V for 3 h [running buffer: 25 mM Tris, 192 mM glycine 1% SDS (w/v)]. Samples were suspended in Laemmli buffer and boiled at 95 °C for 10 min prior to loading. The samples consisted of whole cells (1 OD unit/200 μL Laemmli buffer) or fractionated cells (mixed 3:1 with 4x Laemmli buffer). For Western blotting, proteins were transferred to a nitrocellulose membrane using a semidry Trans-Blot SD cell (Bio-Rad) for 30 min at 15 V. The membrane was then blocked for 1–2 h or overnight in 5% (w/v) nonfat milk (PanReac AppliChem) in Tris-buffered saline (TBS) (50 mM Tris pH 7.4, 200 mM NaCl). MalE was detected using the HisProbe-HRP conjugate (15165, Thermo Scientific) at a dilution of 1:10,000 in TBST (TBS supplemented with 1 mL Tween/L TBS) for 1 to 2 h. The membrane was covered with SuperSignal West Pico PLUS chemiluminescent substrate (Thermo Scientific), and the chemiluminescent signal was captured using an Azure c600 imaging system (Azure Biosystems). GroEL was detected using polyclonal antisera raised to GroEL (NBP2-89011, Novus Biologicals) and a Cy3-labeled secondary antibody (28901106, Cytiva). The fluorescent signal was captured using an Azure c600 imaging system (Azure Biosystems).

## Supporting Information Available

Data that were mentioned in the paper but not shown and data of plasmids, primers, and protein sequences used in the study (PDF)
