# Supplementary material for: Biosensor that Detects Stress Caused by Periplasmic Proteins
Source: ACS Synth Biol. 2024 Apr 27;13(5):1477–91. doi: 10.1021/acssynbio.3c00720 (PMC11106774; doi:10.1021/acssynbio.3c00720)
Supplement: Supplementary file 1 — sb3c00720_si_001.pdf [file sb3c00720_si_001.pdf]

Supporting information

Biosensor that Detects Stress Caused by Periplasmic Proteins

Alister J. Cumming, Diana Khananisho, Mateusz Balka,

Nicklas Liljestrand and Daniel O. Daley

Department of Biochemistry and Biophysics, Stockholm University, SE-19468, Sweden.

Address correspondence to DOD (+46 8 162 910, ddaley@dbb.su.se)

Table S1. A list of primers used in the study

Primer	Purpose	Sequence 5' - 3'
P1	pBAD FwD (MalE)	GAAGGAGATATACATACCCATGAAAATAA
P2	pBAD His ReV (MalE)	GTGGTGATGATGATGCTTGGTGATAC
P3	MalE FwD	GAAATAATTTTGTTTAACTTTAAGAAGGAGATATACATACCCATGAAAATAAAAACAGGTG CACGC
P4	MalE ReV	GACCGTAAGCTTTCAGTGGTGGTGGTGGTGATGATGATGCTTGGTGATACGAGTCTGCG
P5	MalE31 mut FwD	AAAGATACCGATCCTAAAGTCACCGTTGAGCATC
P6	MalE31 mut ReV	AGGATCGGTATCTTTCTCGAATTTCTTACCGACTTCAG
P7	MalEΔC FwD	AAAGAGCTGGCGGCATCATCATCACCACCACCACCAC
P8	MalEΔC ReV	CGCCAGCTCTTTGTTCGGACTGGCGGCGTTAA
P9	malEdC FS fix F	GAACAAAGAGCTGGCGCATCATCATCACCACCACCACC
P10	malEdC FS fix R	CGCCAGCTCTTTGTTCGGACTGGCGG
P11	malEΔSS FwD	CATGGGTATGTATATCTCCTTCTTAAAGTTAAACAAAATTATTTC
P12	malEΔSS Rev	TACATACCCATGAAAATCGAAGAAGGTAAACTGG
P13	AmpR TIR amp20 Fwd	CAATAATATTGAAAAAGGGGATGTATGAGTATTCAACATTTCCGTGTCGCCC
P14	AmpR TIR mut Rev 1	CCTTTTTCAATATTATTGAAGCATTTATC
P15	Fwd_GFP_rep_AN	ATGCGTAAAGGAGAAGAACTTTTCAC
P16	Rev_GFP_rep_AN	TTAATTAAAGGCATCAAATAAAACGAAAGG
P17	pspA Rev	CTTCTCCTTTACGCATAATGTTGTCCTCTTGATTTCTGCG
P18	pspA FwD	CGTTTTATTTGATGCCTGAAAGCTGTTCGCCTCACC
P19	HdeA Rev	CTTCTCCTTTACGCATCGTAATATCCTCAACTATAAAGTGAAAG
P20	HdeA FwD	CGTTTTATTTGATGCCTGAAAATCCCCTGCTATCAATCTATG
P21	RprA FwD	CTTCTCCTTTACGCATCGTGTAATGGCTGCATGAAATAAACAATAAAA
P22	RprA Rev	TATTTGATGCCTTTAATTAAGTTTTTAAACTAATGAGACGAATCTGATCGAC
P23	CpxP FwD	TATTTGATGCCTTTAATTAACGCTAATCCATGACTTTACGTTGTTTTACAC
P24	CpxP Rev	CTTCTCCTTTACGCATCATTTGCTCCCAAAATCTTTCTGTCGC
P25	RpoE FwD	TATTTGATGCCTTTAATTAACAACACGTCACATGAATGTTCAGGG
P26	RpoE Rev	CTTCTCCTTTACGCATCCGAGGTAAAGTCTCCCCAAACC
P27	ASV BB fwD	AGGCCTGCAGCAAACGACGAAAAC
P28	pCpxP BB Rev	CATTTGCTCCCAAAATCTTTCTGTCGCG
P29	CpxP mCherry FwD	ATTTTGGGAGCAAATGATGAGCAAGGGCGAGGAGG
P30	CpxP mCherry Rev	GTTTGCTGCAGGCCTCTTGTACAGCTCGTCCATGCC
P31	cpxp mch flip Fwd	CGTTTTATTTGATGCCTTTAAACTGATGCAGCGTAGTTTTCGTCG
P32	cpxp mch flip Rev	CAGATGAATTTTAATTAACGCTAATCCATGACTTTACGTTGTTTTAC

P33	RepBB cpxp mch in FwD	TTAATTAAAATTCATCTGTTGATCGTGGGTGTTGG
P34	RepBB cpxp mch in ReV	AGGCATCAAATAAAACGAAAGGCTCAGTCG
P35	TsecG FwD	GATCAACAGATGAATTTTAAAAGTCCCGTCCTCGGTACCAAATTC
P36	TsecG ReV	GTCATGGATTAGCGTTAACCTCAGACCAGGACCAAAACGAAAAAAG
P37	BB Dual Term FwD	TTAACGCTAATCCATGACTTTACG
P38	BB Dual Term Rev	TTAAAATTCATCTGTTGATCGTGGG
P39	pCpxPmCh D4 FwD	CAGAAAGATTTTGGGAGATTATTATGTCGAAAGGCGAGGAGGATAACATGGCC
P40	pCpxPmCh Rev	CTCCCAAAATCTTTCTGTCGCGATTC
P41	pCpxPmCh TIROp	CAGAAAGATTTTGGGAGNNNNNNATGTCNAARGGCGAGGAGGATAACATGGCC

Table S2. A list of coding sequences used in this study. Regions encoding signal peptides are marked in bold font. Poly-histidine purification tags are shaded grey, TEV protease recognition sequences in gold and 3xFLAG tags in salmon. Regions encoding terminator sequences are in lower case font. Regions encoding fluorescent proteins are marked in green (GFP) or red (mCherry). Promoter regions are underlined.

preMalE

ATGAAAATAAAAACAGGTGCACGCATCCTCGCATTATCCGCATTAACGACGATGATGTTTTCCGCCTCGGCTCTCGCCAAAATCGAAG
AAGGTAAACTGGTAATCTGGATTAACGGCGATAAAGGCTATAACGGTCTCGCTGAAGTCGGTAAGAAATTCGAGAAAGATACCGGAAT
TAAAGTCACCGTTGAGCATCCGGATAAACTGGAAGAGAAATTCCCACAGGTTGCGGCAACTGGCGATGGCCCTGACATTATCTTCTGGG
CACACGACCGCTTTGGTGGCTACGCTCAATCTGGCCTGTTGGCTGAAATCACCCCGGACAAAGCGTTCCAGGACAAGCTGTATCCGTTTA
CCTGGGATGCCGTACGTTACAACGGCAAGCTGATTGCTTACCCGATCGCTGTTGAAGCGTTATCGCTGATTTATAACAAAGATCTGCTGC
CGAACCCGCCAAAAACCTGGGAAGAGATCCCGGCGCTGGATAAAGAACTGAAAGCGAAAGGTAAGAGCGCGCTGATGTTCAACCTGC
AAGAACCGTACTTCACCTGGCCGCTGATTGCTGCTGACGGGGGTTATGCGTTCAAGTATGAAAACGGCAAGTACGACATTAAAGACGT
GGGCGTGGATAACGCTGGCGCGAAAGCGGGTCTGACCTTCCTGGTTGACCTGATTAAAAACAAACACATGAATGCAGACACCGATTAC
TCCATCGCAGAAGCTGCCTTTAATAAAGGCGAAACAGCGATGACCATCAACGGCCCGTGGGCATGGTCCAACATCGACACCAGCAAAG
TGAATTATGGTGTAACGGTACTGCCGACCTTCAAGGGTCAACCATCCAAACCGTTCGTTGGCGTGCTGAGCGCAGGTATTAACGCCGCC
AGTCCGAACAAAGAGCTGGCGAAAGAGTTCCTCGAAAACTATCTGCTGACTGATGAAGGTCTGGAAGCGGTTAATAAAGACAAACCGC
TGGGTGCCGTAGCGCTGAAGTCTTACGAGGAAGAGTTGGCGAAAGATCCACGTATTGCCGCCACCATGGAAAACGCCCAGAAAGGTGA
AATCATGCCGAACATCCCGCAGATGTCCGCTTTCTGGTATGCCGTGCGTACTGCGGTGATCAACGCCGCCAGCGGTCGTCAGACTGTCG
ATGAAGCCCTGAAAGACGCGCAGACTCGTATCACCAAGCATCATCATCACCACCACCACCACTGA

preMalEΔC

ATGAAAATAAAAACAGGTGCACGCATCCTCGCATTATCCGCATTAACGACGATGATGTTTTCCGCCTCGGCTCTCGCCAAAATCGAAG
AAGGTAAACTGGTAATCTGGATTAACGGCGATAAAGGCTATAACGGTCTCGCTGAAGTCGGTAAGAAATTCGAGAAAGATACCGGAAT
TAAAGTCACCGTTGAGCATCCGGATAAACTGGAAGAGAAATTCCCACAGGTTGCGGCAACTGGCGATGGCCCTGACATTATCTTCTGGG
CACACGACCGCTTTGGTGGCTACGCTCAATCTGGCCTGTTGGCTGAAATCACCCCGGACAAAGCGTTCCAGGACAAGCTGTATCCGTTTA
CCTGGGATGCCGTACGTTACAACGGCAAGCTGATTGCTTACCCGATCGCTGTTGAAGCGTTATCGCTGATTTATAACAAAGATCTGCTGC
CGAACCCGCCAAAAACCTGGGAAGAGATCCCGGCGCTGGATAAAGAACTGAAAGCGAAAGGTAAGAGCGCGCTGATGTTCAACCTGC
AAGAACCGTACTTCACCTGGCCGCTGATTGCTGCTGACGGGGGTTATGCGTTCAAGTATGAAAACGGCAAGTACGACATTAAAGACGT
GGGCGTGGATAACGCTGGCGCGAAAGCGGGTCTGACCTTCCTGGTTGACCTGATTAAAAACAAACACATGAATGCAGACACCGATTAC
TCCATCGCAGAAGCTGCCTTTAATAAAGGCGAAACAGCGATGACCATCAACGGCCCGTGGGCATGGTCCAACATCGACACCAGCAAAG
TGAATTATGGTGTAACGGTACTGCCGACCTTCAAGGGTCAACCATCCAAACCGTTCGTTGGCGTGCTGAGCGCAGGTATTAACGCCGCC
AGTCCGAACAAAGAGCTGGCGCATCATCATCACCACCACCACCACTAG

preMalE31

ATGAAAATAAAAACAGGTGCACGCATCCTCGCATTATCCGCATTAACGACGATGATGTTTTCCGCCTCGGCTCTCGCCAAAATCGAAG
AAGGTAAACTGGTAATCTGGATTAACGGCGATAAAGGCTATAACGGTCTCGCTGAAGTCGGTAAGAAATTCGAGAAAGATACCGATCC
TAAAGTCACCGTTGAGCATCCGGATAAACTGGAAGAGAAATTCCCACAGGTTGCGGCAACTGGCGATGGCCCTGACATTATCTTCTGGG
CACACGACCGCTTTGGTGGCTACGCTCAATCTGGCCTGTTGGCTGAAATCACCCCGGACAAAGCGTTCCAGGACAAGCTGTATCCGTTTA
CCTGGGATGCCGTACGTTACAACGGCAAGCTGATTGCTTACCCGATCGCTGTTGAAGCGTTATCGCTGATTTATAACAAAGATCTGCTGC
CGAACCCGCCAAAAACCTGGGAAGAGATCCCGGCGCTGGATAAAGAACTGAAAGCGAAAGGTAAGAGCGCGCTGATGTTCAACCTGC
AAGAACCGTACTTCACCTGGCCGCTGATTGCTGCTGACGGGGGTTATGCGTTCAAGTATGAAAACGGCAAGTACGACATTAAAGACGT
GGGCGTGGATAACGCTGGCGCGAAAGCGGGTCTGACCTTCCTGGTTGACCTGATTAAAAACAAACACATGAATGCAGACACCGATTAC
TCCATCGCAGAAGCTGCCTTTAATAAAGGCGAAACAGCGATGACCATCAACGGCCCGTGGGCATGGTCCAACATCGACACCAGCAAAG
TGAATTATGGTGTAACGGTACTGCCGACCTTCAAGGGTCAACCATCCAAACCGTTCGTTGGCGTGCTGAGCGCAGGTATTAACGCCGCC
AGTCCGAACAAAGAGCTGGCGAAAGAGTTCCTCGAAAACTATCTGCTGACTGATGAAGGTCTGGAAGCGGTTAATAAAGACAAACCGC
TGGGTGCCGTAGCGCTGAAGTCTTACGAGGAAGAGTTGGCGAAAGATCCACGTATTGCCGCCACCATGGAAAACGCCCAGAAAGGTGA
AATCATGCCGAACATCCCGCAGATGTCCGCTTTCTGGTATGCCGTGCGTACTGCGGTGATCAACGCCGCCAGCGGTCGTCAGACTGTCG
ATGAAGCCCTGAAAGACGCGCAGACTCGTATCACCAAGCATCATCATCACCACCACCACCACTGA

ΔssMalE

ATGAAAATCGAAGAAGGTAAACTGGTAATCTGGATTAACGGCGATAAAGGCTATAACGGTCTCGCTGAAGTCGGTAAGAAATTCGAGA
AAGATACCGGAATTAAAGTCACCGTTGAGCATCCGGATAAACTGGAAGAGAAATTCCCACAGGTTGCGGCAACTGGCGATGGCCCTGA
CATTATCTTCTGGGCACACGACCGCTTTGGTGGCTACGCTCAATCTGGCCTGTTGGCTGAAATCACCCCGGACAAAGCGTTCCAGGACAA
GCTGTATCCGTTTACCTGGGATGCCGTACGTTACAACGGCAAGCTGATTGCTTACCCGATCGCTGTTGAAGCGTTATCGCTGATTTATAA
CAAAGATCTGCTGCCGAACCCGCCAAAAACCTGGGAAGAGATCCCGGCGCTGGATAAAGAACTGAAAGCGAAAGGTAAGAGCGCGCT
GATGTTCAACCTGCAAGAACCGTACTTCACCTGGCCGCTGATTGCTGCTGACGGGGGTTATGCGTTCAAGTATGAAAACGGCAAGTACG
ACATTAAAGACGTGGGCGTGGATAACGCTGGCGCGAAAGCGGGTCTGACCTTCCTGGTTGACCTGATTAAAAACAAACACATGAATGC
AGACACCGATTACTCCATCGCAGAAGCTGCCTTTAATAAAGGCGAAACAGCGATGACCATCAACGGCCCGTGGGCATGGTCCAACATCG
ACACCAGCAAAGTGAATTATGGTGTAACGGTACTGCCGACCTTCAAGGGTCAACCATCCAAACCGTTCGTTGGCGTGCTGAGCGCAGGT
ATTAACGCCGCCAGTCCGAACAAAGAGCTGGCGAAAGAGTTCCTCGAAAACTATCTGCTGACTGATGAAGGTCTGGAAGCGGTTAATA
AAGACAAACCGCTGGGTGCCGTAGCGCTGAAGTCTTACGAGGAAGAGTTGGCGAAAGATCCACGTATTGCCGCCACCATGGAAAACGC
CCAGAAAGGTGAAATCATGCCGAACATCCCGCAGATGTCCGCTTTCTGGTATGCCGTGCGTACTGCGGTGATCAACGCCGCCAGCGGTC
GTCAGACTGTCGATGAAGCCCTGAAAGACGCGCAGACTCGTATCACCAAGCATCATCATCACCACCACCACCACTGA

ΔssMalEΔC

ATGAAAATCGAAGAAGGTAAACTGGTAATCTGGATTAACGGCGATAAAGGCTATAACGGTCTCGCTGAAGTCGGTAAGAAATTCGAGA
AAGATACCGGAATTAAAGTCACCGTTGAGCATCCGGATAAACTGGAAGAGAAATTCCCACAGGTTGCGGCAACTGGCGATGGCCCTGA
CATTATCTTCTGGGCACACGACCGCTTTGGTGGCTACGCTCAATCTGGCCTGTTGGCTGAAATCACCCCGGACAAAGCGTTCCAGGACAA
GCTGTATCCGTTTACCTGGGATGCCGTACGTTACAACGGCAAGCTGATTGCTTACCCGATCGCTGTTGAAGCGTTATCGCTGATTTATAA
CAAAGATCTGCTGCCGAACCCGCCAAAAACCTGGGAAGAGATCCCGGCGCTGGATAAAGAACTGAAAGCGAAAGGTAAGAGCGCGCT
GATGTTCAACCTGCAAGAACCGTACTTCACCTGGCCGCTGATTGCTGCTGACGGGGGTTATGCGTTCAAGTATGAAAACGGCAAGTACG
ACATTAAAGACGTGGGCGTGGATAACGCTGGCGCGAAAGCGGGTCTGACCTTCCTGGTTGACCTGATTAAAAACAAACACATGAATGC
AGACACCGATTACTCCATCGCAGAAGCTGCCTTTAATAAAGGCGAAACAGCGATGACCATCAACGGCCCGTGGGCATGGTCCAACATCG
ACACCAGCAAAGTGAATTATGGTGTAACGGTACTGCCGACCTTCAAGGGTCAACCATCCAAACCGTTCGTTGGCGTGCTGAGCGCAGGT
ATTAACGCCGCCAGTCCGAACAAAGAGCTGGCGCATCATCATCACCACCACCACCACTAG

ΔssMalE31

ATGAAAATCGAAGAAGGTAAACTGGTAATCTGGATTAACGGCGATAAAGGCTATAACGGTCTCGCTGAAGTCGGTAAGAAATTCGAGA
AAGATACCGATCCTAAAGTCACCGTTGAGCATCCGGATAAACTGGAAGAGAAATTCCCACAGGTTGCGGCAACTGGCGATGGCCCTGA
CATTATCTTCTGGGCACACGACCGCTTTGGTGGCTACGCTCAATCTGGCCTGTTGGCTGAAATCACCCCGGACAAAGCGTTCCAGGACAA
GCTGTATCCGTTTACCTGGGATGCCGTACGTTACAACGGCAAGCTGATTGCTTACCCGATCGCTGTTGAAGCGTTATCGCTGATTTATAA
CAAAGATCTGCTGCCGAACCCGCCAAAAACCTGGGAAGAGATCCCGGCGCTGGATAAAGAACTGAAAGCGAAAGGTAAGAGCGCGCT
GATGTTCAACCTGCAAGAACCGTACTTCACCTGGCCGCTGATTGCTGCTGACGGGGGTTATGCGTTCAAGTATGAAAACGGCAAGTACG
ACATTAAAGACGTGGGCGTGGATAACGCTGGCGCGAAAGCGGGTCTGACCTTCCTGGTTGACCTGATTAAAAACAAACACATGAATGC
AGACACCGATTACTCCATCGCAGAAGCTGCCTTTAATAAAGGCGAAACAGCGATGACCATCAACGGCCCGTGGGCATGGTCCAACATCG
ACACCAGCAAAGTGAATTATGGTGTAACGGTACTGCCGACCTTCAAGGGTCAACCATCCAAACCGTTCGTTGGCGTGCTGAGCGCAGGT
ATTAACGCCGCCAGTCCGAACAAAGAGCTGGCGAAAGAGTTCCTCGAAAACTATCTGCTGACTGATGAAGGTCTGGAAGCGGTTAATA
AAGACAAACCGCTGGGTGCCGTAGCGCTGAAGTCTTACGAGGAAGAGTTGGCGAAAGATCCACGTATTGCCGCCACCATGGAAAACGC
CCAGAAAGGTGAAATCATGCCGAACATCCCGCAGATGTCCGCTTTCTGGTATGCCGTGCGTACTGCGGTGATCAACGCCGCCAGCGGTC
GTCAGACTGTCGATGAAGCCCTGAAAGACGCGCAGACTCGTATCACCAAGCATCATCATCACCACCACCACCACTGA

[Pr_{ibpA}-gfp_{ASV}]

atttgtcctactcaggagagcgttcaccgacaaacaacagataaaacgaaaggcccagtctttcgactgagcctttcgttttatttgATGCCTTTAATTAAAATTCATCTG
TTGATCGTGGGTGTTGGCCTGATGAGTTATAGCGATCCCTTGCTGAAAATAACATCATCATTACGTCGCACTGTGGCGGCTATCGCACTT
TAACGTTTCGTGCTGCCCCCTCAGTCTATGCAATAGACCATAAACTGCAAAAAAAAGTCCGCTGATAAGGCTTGAAAAGTTCATTTCCAG
ACCCATTTTTACATCGTAGCCGATGAGGACGCGCCTGATGGGTGTTCTGGCTACCTGACCTGTCCATTGTGGAAGGTCTTACATTCTCGC
TGATTTCAGGAGCTATCTAGAATGCGTAAAGGAGAAGAACTTTTCACTGGAGTTGTCCCAATTCTTGTTGAATTAGATGGTGATGTTAAT
GGGCACAAATTTTCTGTCAGTGGAGAGGGTGAAGGTGATGCAACATACGGAAAACTTACCCTTAAATTTATTTGCACTACTGGAAAACT
ACCTGTTCCATGGCCAACACTTGTCACTACTTTCGGTTATGGTGTTCAATGCTTTGCGAGATACCCAGATCATATGAAACAGCATGACTTT

TTCAAGAGTGCCATGCCCGAAGGTTATGTACAGGAAAGAACTATATTTTTCAAAGATGACGGGAACTACAAGACACGTGCTGAAGTCAA
GTTTGAAGGTGATACCCTTGTTAATAGAATCGAGTTAAAAGGTATTGATTTTAAAGAAGATGGAAACATTCTTGGACACAAATTGGAAT
ACAACTATAACTCACACAATGTATACATCATGGCAGACAAACAAAAGAATGGAATCAAAGTTAACTTCAAAATTAGACACAACATTGAA
GATGGAAGCGTTCAACTAGCAGACCATTATCAACAAAATACTCCAATTGGCGATGGCCCTGTCCTTTTACCAGACAACCATTACCTGTCC
ACACAATCTGCCCTTTCGAAAGATCCCAACGAAAAGAGAGACCACATGGTCCTTCTTGAGTTTGTAACAGCTGCTGGGATTACACATGGC
ATGGATGAACTATACAAAAGGCCTGCAGCAAACGACGAAAACTACGCTGCATCAGTTTAAACTAGTCTTGgactcctgttgatagatccagtaat
gacctcagaactccatctggatttgttcagaacgctcggttgccgccgggcgtttttttattggtgagaat

[P$_{cpxP}$-gfp$_{ASV}$] (Cpx)

atttgtcctactcaggagagcgttcaccgacaaacaacagataaaacgaaaggcccagtctttcgactgagcctttcgttttatttgATGCCTTTAATTAACGCTAATCCA
TGACTTTACGTTGTTTTACACCCCCTGACGCATGTTTGCAGCCTGAATCGTAAACTCTCTATCGTTGAATCGCGACAGAAAGATTTTGGGA
GCAAATGATGCGTAAAGGAGAAGAACTTTTCACTGGAGTTGTCCCAATTCTTGTTGAATTAGATGGTGATGTTAATGGGCACAAATTTTC
TGTCAGTGGAGAGGGTGAAGGTGATGCAACATACGGAAAACTTACCCTTAAATTTATTTGCACTACTGGAAAACTACCTGTTCCATGGC
CAACACTTGTCACTACTTTCGGTTATGGTGTTCAATGCTTTGCGAGATACCCAGATCATATGAAACAGCATGACTTTTTCAAGAGTGCCAT
GCCCGAAGGTTATGTACAGGAAAGAACTATATTTTTCAAAGATGACGGGAACTACAAGACACGTGCTGAAGTCAAGTTTGAAGGTGAT
ACCCTTGTTAATAGAATCGAGTTAAAAGGTATTGATTTTAAAGAAGATGGAAACATTCTTGGACACAAATTGGAATACAACTATAACTCA
CACAATGTATACATCATGGCAGACAAACAAAAGAATGGAATCAAAGTTAACTTCAAAATTAGACACAACATTGAAGATGGAAGCGTTCA
ACTAGCAGACCATTATCAACAAAATACTCCAATTGGCGATGGCCCTGTCCTTTTACCAGACAACCATTACCTGTCCACACAATCTGCCCTT
TCGAAAGATCCCAACGAAAAGAGAGACCACATGGTCCTTCTTGAGTTTGTAACAGCTGCTGGGATTACACATGGCATGGATGAACTATA
CAAAAGGCCTGCAGCAAACGACGAAAACTACGCTGCATCAGTTTAAACTAGTCTTGgactcctgttgatagatccagtaatgacctcagaactccatctg
gatttgttcagaacgctcggttgccgccgggcgtttttttattggtgagaat

[P$_{cpxP}$-mcherry$_{ASV}$] (Cpx)

atttgtcctactcaggagagcgttcaccgacaaacaacagataaaacgaaaggcccagtctttcgactgagcctttcgttttatttgATGCCTTTAATTAACGCTAATCCA
TGACTTTACGTTGTTTTACACCCCCTGACGCATGTTTGCAGCCTGAATCGTAAACTCTCTATCGTTGAATCGCGACAGAAAGATTTTGGGA
GCAAATGATGAGCAAGGGCGAGGAGGATAACATGGCCATCATCAAGGAGTTCATGCGCTTCAAGGTGCACATGGAGGGCTCCGTGAA
CGGCCACGAGTTCGAGATCGAGGGCGAGGGCGAGGGCCGCCCCTACGAGGGCACCCAGACCGCCAAGCTGAAGGTGACCAAGGGTG
GCCCCCTGCCCTTCGCCTGGGACATCCTGTCCCCTCAGTTCATGTACGGCTCCAAGGCCTACGTGAAGCACCCCGCCGACATCCCCGACT
ACTTGAAGCTGTCCTTCCCCGAGGGCTTCAAGTGGGAGCGCGTGATGAACTTCGAGGACGGCGGCGTGGTGACCGTGACCCAGGACTC
CTCCCTGCAGGACGGCGAGTTCATCTACAAGGTGAAGCTGCGCGGCACCAACTTCCCCTCCGACGGCCCCGTAATGCAGAAGAAGACCA
TGGGCTGGGAGGCCTCCTCCGAGCGGATGTACCCCGAGGACGGCGCCCTGAAGGGCGAGATCAAGCAGAGGCTGAAGCTGAAGGAC
GGCGGCCACTACGACGCTGAGGTCAAGACCACCTACAAGGCCAAGAAGCCCGTGCAGCTGCCCGGCGCCTACAACGTCAACATCAAGT
TGGACATCACCTCCCACAACGAGGACTACACCATCGTGGAACAGTACGAACGCGCCGAGGGCCGCCACTCCACCGGCGGCATGGACGA
GCTGTACAAGAGGCCTGCAGCAAACGACGAAAACTACGCTGCATCAGTTTAAACTAGTCTTGgactcctgttgatagatccagtaatgacctcagaa
ctccatctggatttgttcagaacgctcggttgccgccgggcgtttttttattggtgagaat

[P$_{cpxP}$-mcherry$_{ASV}$]^{OPT} (Cpx)

atttgtcctactcaggagagcgttcaccgacaaacaacagataaaacgaaaggcccagtctttcgactgagcctttcgttttatttgATGCCTTTAATTAACGCTAATCCA
TGACTTTACGTTGTTTTACACCCCCTGACGCATGTTTGCAGCCTGAATCGTAAACTCTCTATCGTTGAATCGCGACAGAAAGATTTTGGGA
GATTATTATGTCGAAAGGCGAGGAGGATAACATGGCCATCATCAAGGAGTTCATGCGCTTCAAGGTGCACATGGAGGGCTCCGTGAAC
GGCCACGAGTTCGAGATCGAGGGCGAGGGCGAGGGCCGCCCCTACGAGGGCACCCAGACCGCCAAGCTGAAGGTGACCAAGGGTGG
CCCCCTGCCCTTCGCCTGGGACATCCTGTCCCCTCAGTTCATGTACGGCTCCAAGGCCTACGTGAAGCACCCCGCCGACATCCCCGACTA
CTTGAAGCTGTCCTTCCCCGAGGGCTTCAAGTGGGAGCGCGTGATGAACTTCGAGGACGGCGGCGTGGTGACCGTGACCCAGGACTCC
TCCCTGCAGGACGGCGAGTTCATCTACAAGGTGAAGCTGCGCGGCACCAACTTCCCCTCCGACGGCCCCGTAATGCAGAAGAAGACCAT
GGGCTGGGAGGCCTCCTCCGAGCGGATGTACCCCGAGGACGGCGCCCTGAAGGGCGAGATCAAGCAGAGGCTGAAGCTGAAGGACG
GCGGCCACTACGACGCTGAGGTCAAGACCACCTACAAGGCCAAGAAGCCCGTGCAGCTGCCCGGCGCCTACAACGTCAACATCAAGTT
GGACATCACCTCCCACAACGAGGACTACACCATCGTGGAACAGTACGAACGCGCCGAGGGCCGCCACTCCACCGGCGGCATGGACGAG
CTGTACAAGAGGCCTGCAGCAAACGACGAAAACTACGCTGCATCAGTTTAAACTAGTCTTGgactcctgttgatagatccagtaatgacctcagaact
ccatctggatttgttcagaacgctcggttgccgccgggcgtttttttattggtgagaat

[P_{rpoE}-gfp_{ASV}] (σ^{E})

atttgtcctactcaggagagcgttcaccgacaaacaacagataaaacgaaaggcccagtctttcgactgagcctttcgttttatttgATGCCTTTAATTAACAACACGTCA
CATGAATGTTCAGGGAGAGTATTCATTTTCTTTGTTTAATTTACTAAACATGGTTTGGTCAGCATAGCATCATGTTGTGCGAATAAACACC
TGCTATTTTAATATTTGTTACAGTGGCTAAACACGCTGACGCAGGGCGGCGAGAAAAAGAGAAGTTACTGGCTGGTGGAGGATTAGGT
GGTGAAATAAAAAGGCCGTTGGGTTACTCTTCAGGCAGTTAAATGGGCATTTCTACACAGATAATGCGATGTTCAGATTCTGTAGACTT
ATAATGATAGATAATGATCCGTCTACAGCATGACAAACAAAAACAGATGCGTTACGGAACTTTACAAAAACGAGACACTCTAACCCTTT
GCTTGCTCAAATTGCAGCTAATGGAGTGGCGTTTCGATAGCGCGTGGAAATTTGGTTTGGGGAGACTTTACCTCGGATGCGTAAAGGA
GAAGAACTTTTCACTGGAGTTGTCCCAATTCTTGTTGAATTAGATGGTGATGTTAATGGGCACAAATTTTCTGTCAGTGGAGAGGGTGA
AGGTGATGCAACATACGGAAAACTTACCCTTAAATTTATTTGCACTACTGGAAAACTACCTGTTCCATGGCCAACACTTGTCACTACTTTC
GGTTATGGTGTTCAATGCTTTGCGAGATACCCAGATCATATGAAACAGCATGACTTTTTCAAGAGTGCCATGCCCGAAGGTTATGTACAG
GAAAGAACTATATTTTTCAAAGATGACGGGAACTACAAGACACGTGCTGAAGTCAAGTTTGAAGGTGATACCCTTGTTAATAGAATCGA
GTTAAAAGGTATTGATTTTAAAGAAGATGGAAACATTCTTGGACACAAATTGGAATACAACTATAACTCACACAATGTATACATCATGGC
AGACAAACAAAAGAATGGAATCAAAGTTAACTTCAAAATTAGACACAACATTGAAGATGGAAGCGTTCAACTAGCAGACCATTATCAAC
AAAATACTCCAATTGGCGATGGCCCTGTCCTTTTACCAGACAACCATTACCTGTCCACACAATCTGCCCTTTCGAAAGATCCCAACGAAAA
GAGAGACCACATGGTCCTTCTTGAGTTTGTAACAGCTGCTGGGATTACACATGGCATGGATGAACTATACAAAAGGCCTGCAGCAAACG
ACGAAAACTACGCTGCATCAGTTTAAACTAGTCTTGgactcctgttgatagatccagtaatgacctcagaactccatctggatttgttcagaacgctcggttgccgc
cgggcgtttttattggtgagaat

[P_{rprA}-gfp_{ASV}] (Rcs)

atttgtcctactcaggagagcgttcaccgacaaacaacagataaaacgaaaggcccagtctttcgactgagcctttcgttttatttgATGCCTTTAATTAAGTTTTTAAAC
TAATGAGACGAATCTGATCGACGCAAAAAGTCCGTATGCCTACTATTAGCTCACGGTTATAAATCAACATATTGATTTATAAGCATGGAA
ATCCCCTGAGTGAAACAACGAATTGCTGTGTGTAGTCTTTGCCCATCTCCCACGATGGGCTTTTTTTTAACATTTTTCCGCATCGCTACCTC
GCCCCTCACTCTTTCCAATAATGTTGCACAAGATACTGTGACTCTCTTCAAGCCGTCAGCAGGCTGATAATGTTATTTATATTTTATTGTTT
ATTTCATGCAGCCATTACACGATGCGTAAAGGAGAAGAACTTTTCACTGGAGTTGTCCCAATTCTTGTTGAATTAGATGGTGATGTTAAT
GGGCACAAATTTTCTGTCAGTGGAGAGGGTGAAGGTGATGCAACATACGGAAAACTTACCCTTAAATTTATTTGCACTACTGGAAAACT
ACCTGTTCCATGGCCAACACTTGTCACTACTTTCGGTTATGGTGTTCAATGCTTTGCGAGATACCCAGATCATATGAAACAGCATGACTTT
TTCAAGAGTGCCATGCCCGAAGGTTATGTACAGGAAAGAACTATATTTTTCAAAGATGACGGGAACTACAAGACACGTGCTGAAGTCAA
GTTTGAAGGTGATACCCTTGTTAATAGAATCGAGTTAAAAGGTATTGATTTTAAAGAAGATGGAAACATTCTTGGACACAAATTGGAAT
ACAACTATAACTCACACAATGTATACATCATGGCAGACAAACAAAAGAATGGAATCAAAGTTAACTTCAAAATTAGACACAACATTGAA
GATGGAAGCGTTCAACTAGCAGACCATTATCAACAAAATACTCCAATTGGCGATGGCCCTGTCCTTTTACCAGACAACCATTACCTGTCC
ACACAATCTGCCCTTTCGAAAGATCCCAACGAAAAGAGAGACCACATGGTCCTTCTTGAGTTTGTAACAGCTGCTGGGATTACACATGGC
ATGGATGAACTATACAAAAGGCCTGCAGCAAACGACGAAAACTACGCTGCATCAGTTTAAACTAGTCTTGgactcctgttgatagatccagtaat
gacctcagaactccatctggatttgttcagaacgctcggttgccgccgggcgtttttattggtgagaat

[P_{pspA}-gfp_{ASV}] (Psp)

atttgtcctactcaggagagcgttcaccgacaaacaacagataaaacgaaaggcccagtctttcgactgagcctttcgttttatttgATGCCTGAAAGCTGTTCGCCTCA
CCAAGTAAATTATCTTTGTATTCTGCCATGATGAAATTCGCCACTTGTTAGTGTAATTCGCTAACTCATCCTGGCATGTTGCTGTTGATTCT
TCAATCAGATCTTTATAAATCAAAAAGATAAAAAATTGGCACGCAAATTGTATTAACAGTTCAGCAGGACAATCCTGAACGCAGAAATCA
AGAGGACAACATTATGCGTAAAGGAGAAGAACTTTTCACTGGAGTTGTCCCAATTCTTGTTGAATTAGATGGTGATGTTAATGGGCACA
AATTTTCTGTCAGTGGAGAGGGTGAAGGTGATGCAACATACGGAAAACTTACCCTTAAATTTATTTGCACTACTGGAAAACTACCTGTTC
CATGGCCAACACTTGTCACTACTTTCGGTTATGGTGTTCAATGCTTTGCGAGATACCCAGATCATATGAAACAGCATGACTTTTTCAAGA
GTGCCATGCCCGAAGGTTATGTACAGGAAAGAACTATATTTTTCAAAGATGACGGGAACTACAAGACACGTGCTGAAGTCAAGTTTGAA
GGTGATACCCTTGTTAATAGAATCGAGTTAAAAGGTATTGATTTTAAAGAAGATGGAAACATTCTTGGACACAAATTGGAATACAACTAT
AACTCACACAATGTATACATCATGGCAGACAAACAAAAGAATGGAATCAAAGTTAACTTCAAAATTAGACACAACATTGAAGATGGAAG
CGTTCAACTAGCAGACCATTATCAACAAAATACTCCAATTGGCGATGGCCCTGTCCTTTTACCAGACAACCATTACCTGTCCACACAATCT
GCCCTTTCGAAAGATCCCAACGAAAAGAGAGACCACATGGTCCTTCTTGAGTTTGTAACAGCTGCTGGGATTACACATGGCATGGATGA
ACTATACAAAAGGCCTGCAGCAAACGACGAAAACTACGCTGCATCAGTTTAAACTAGTCTTGgactcctgttgatagatccagtaatgacctcagaac
tccatctggatttgttcagaacgctcggttgccgccgggcgtttttattggtgagaat

[P_{hdeA}-gfp$_{ASV}$] (acid stress)

atttgtcctactcaggagagcgttcaccgacaaacaacagataaaacgaaaggcccagtctttcgactgagcctttcgttttatttgATGCCTGAAAATCCCCTGCTATCA
ATCTATGCCAAAAACGCGTCTAAGAATGCAGTCGATTTAATAAAAATTTCCTAATTGCAGTATCTGATGCATCTGTAACTCATTGTATTGA
AATAAAAATATCTGATTTTGATATTTTCCATCAACATGACATATACAGAAAACCAGGTTATAACCTCAGTGTCGAAATTGATTCGTGACG
GCTCTTTCACTTTATAGTTGAGGATATTACGATGCGTAAAGGAGAAGAACTTTTCACTGGAGTTGTCCCAATTCTTGTTGAATTAGATGG
TGATGTTAATGGGCACAAATTTTCTGTCAGTGGAGAGGGTGAAGGTGATGCAACATACGGAAAACTTACCCTTAAATTTATTTGCACTAC
TGGAAAACTACCTGTTCCATGGCCAACACTTGTCACTACTTTCGGTTATGGTGTTCAATGCTTTGCGAGATACCCAGATCATATGAAACA
GCATGACTTTTTCAAGAGTGCCATGCCCGAAGGTTATGTACAGGAAAGAACTATATTTTTCAAAGATGACGGGAACTACAAGACACGTG
CTGAAGTCAAGTTTGAAGGTGATACCCTTGTTAATAGAATCGAGTTAAAAGGTATTGATTTTAAAGAAGATGGAAACATTCTTGGACAC
AAATTGGAATACAACTATAACTCACACAATGTATACATCATGGCAGACAAACAAAAGAATGGAATCAAAGTTAACTTCAAAATTAGACA
CAACATTGAAGATGGAAGCGTTCAACTAGCAGACCATTATCAACAAAATACTCCAATTGGCGATGGCCCTGTCCTTTTACCAGACAACCA
TTACCTGTCCACACAATCTGCCCTTTCGAAAGATCCCAACGAAAAGAGAGACCACATGGTCCTTCTTGAGTTTGTAACAGCTGCTGGGAT
TACACATGGCATGGATGAACTATACAAAAGGCCTGCAGCAAACGACGAAAACTACGCTGCATCAGTTTAAACTAGTCTTGgactcctgttgat
agatccagtaatgacctcagaactccatctggatttgttcagaacgctcggttgccgccgggcgtttttttattggtgagaat

[Pr_{ibpA}-gfp$_{ASV}$] / [Pr_{cpxP}-mcherry$_{ASV}$]^{OPT}

atttgtcctactcaggagagcgttcaccgacaaacaacagataaaacgaaaggcccagtctttcgactgagcctttcgttttatttgATGCCTTTAAACTGATGCAGCGT
AGTTTTCGTCGTTTGCTGCAGGCCTCTTGTACAGCTCGTCCATGCCGCCGGTGGAGTGGCGGCCCTCGGCGCGTTCGTACTGTTCCACGA
TGGTGTAGTCCTCGTTGTGGGAGGTGATGTCCAACTTGATGTTGACGTTGTAGGCGCCGGGCAGCTGCACGGGCTTCTTGGCCTTGTAG
GTGGTCTTGACCTCAGCGTCGTAGTGGCCGCCGTCCTTCAGCTTCAGCCTCTGCTTGATCTCGCCCTTCAGGGCGCCGTCCTCGGGGTAC
ATCCGCTCGGAGGAGGCCTCCCAGCCCATGGTCTTCTTCTGCATTACGGGGCCGTCGGAGGGGAAGTTGGTGCCGCGCAGCTTCACCTT
GTAGATGAACTCGCCGTCCTGCAGGGAGGAGTCCTGGGTCACGGTCACCACGCCGCCGTCCTCGAAGTTCATCACGCGCTCCCACTTGA
AGCCCTCGGGGAAGGACAGCTTCAAGTAGTCGGGGATGTCGGCGGGGTGCTTCACGTAGGCCTTGGAGCCGTACATGAACTGAGGGG
ACAGGATGTCCCAGGCGAAGGGCAGGGGGCCACCCTTGGTCACCTTCAGCTTGGCGGTCTGGGTGCCCTCGTAGGGGCGGCCCTCGCC
CTCGCCCTCGATCTCGAACTCGTGGCCGTTCACGGAGCCCTCCATGTGCACCTTGAAGCGCATGAACTCCTTGATGATGGCCATGTTATC
CTCCTCGCCTTTCGACATAATAATCTCCCAAAATCTTTCTGTCGCGATTCAACGATAGAGAGTTTACGATTCAGGCTGCAAACATGCGTCA
GGGGGTGTAAAACAACGTAAAGTCATGGATTAGCGTTAAcctcagaccaggaccaaaacgaaaaaagacgcttttcagcgtctcttttctggaatttggtaccg
aggacgggacttTTAAAATTCATCTGTTGATCGTGGGTGTTGGCCTGATGAGTTATAGCGATCCCTTGCTGAAAATAACATCATCATTACGTC
GCACTGTGGCGGCTATCGCACTTTAACGTTTCGTGCTGCCCCCTCAGTCTATGCAATAGACCATAAACTGCAAAAAAAAGTCCGCTGATA
AGGCTTGAAAAGTTCATTTCCAGACCCATTTTTACATCGTAGCCGATGAGGACGCGCCTGATGGGTGTTCTGGCTACCTGACCTGTCCAT
TGTGGAAGGTCTTACATTCTCGCTGATTTCAGGAGCTATCTAGAATGCGTAAAGGAGAAGAACTTTTCACTGGAGTTGTCCCAATTCTTG
TTGAATTAGATGGTGATGTTAATGGGCACAAATTTTCTGTCAGTGGAGAGGGTGAAGGTGATGCAACATACGGAAAACTTACCCTTAAA
TTTATTTGCACTACTGGAAAACTACCTGTTCCATGGCCAACACTTGTCACTACTTTCGGTTATGGTGTTCAATGCTTTGCGAGATACCCAG
ATCATATGAAACAGCATGACTTTTTCAAGAGTGCCATGCCCGAAGGTTATGTACAGGAAAGAACTATATTTTTCAAAGATGACGGGAAC
TACAAGACACGTGCTGAAGTCAAGTTTGAAGGTGATACCCTTGTTAATAGAATCGAGTTAAAAGGTATTGATTTTAAAGAAGATGGAAA
CATTCTTGGACACAAATTGGAATACAACTATAACTCACACAATGTATACATCATGGCAGACAAACAAAAGAATGGAATCAAAGTTAACTT
CAAAATTAGACACAACATTGAAGATGGAAGCGTTCAACTAGCAGACCATTATCAACAAAATACTCCAATTGGCGATGGCCCTGTCCTTTT
ACCAGACAACCATTACCTGTCCACACAATCTGCCCTTTCGAAAGATCCCAACGAAAAGAGAGACCACATGGTCCTTCTTGAGTTTGTAAC
AGCTGCTGGGATTACACATGGCATGGATGAACTATACAAAAGGCCTGCAGCAAACGACGAAAACTACGCTGCATCAGTTTAAACTAGTC
TTGgactcctgttgatagatccagtaatgacctcagaactccatctggatttgttcagaacgctcggttgccgccgggcgtttttttattggtgagaat

PhoA(evo)$_{ss}$-scFv^{Her2}

ATGAAGCAAAGCACTATTGCACTGGCACTCTTACCGTTACTGTTTACCCCTGTGACAAAAGCCGAAGTGCAGCTGGTCGAATCGGGTG
GCGGATTAGTGCAGCCTGGAGGCTCCTTACGCCTGAGCTGTGCAGCGAGCGGCTTCAACATCAAGGACACCTACATACATTGGGTTCGC
CAAGCTCCGGGCAAAGGTCTGGAGTGGGTTGCTCGTATCTATCCCACTAATGGGTATACACGCTATGCCGATAGCGTGAAAGGCCGGTT
TACCATTAGCGCCGATACGAGCAAGAATACGGCGTATCTGCAGATGAACTCTCTGCGTGCCGAAGATACAGCGGTCTACTACTGCTCTC
GTTGGGGTGGTGACGGGTTTTATGCAATGGACTATTGGGGCCAAGGAACCCTCGTGACGGTTTCCTCAGGCGGAGGTGGTAGTGGTG
GCGGTGGGTCTGGCGGCGGTGGGAGCGACATTCAGATGACGCAGTCACCATCGTCGTTGTCAGCGTCGGTAGGTGATCGCGTCACGAT
TACCTGTCGTGCATCCCAAGATGTGAACACTGCAGTAGCGTGGTACCAGCAGAAACCGGGGAAAGCTCCGAAACTTCTGATTTACTCGG
CGAGTTTCCTGTATAGTGGCGTTCCAAGTCGCTTTAGCGGTTCCCGTTCTGGCACGGATTTCACACTGACCATCTCAAGCTTGCAGCCGG
AAGATTTTGCCACCTATTACTGCCAACAGCACTATACCACTCCTCCGACCTTTGGCCAAGGCACCAAAGTGGAGATCAAACGCCTAGGTG
ATTATAAAGACCATGACGGTGATTATAAAGATCATGACATCGATTACAAGGATGACGATGACAAGGCGGCCGCCCATCATCATCATCAT
CATTAA

PelB$_{ss}$-hGH

ATGAAATACCTGCTGCCGACCGCTGCTGCTGGTCTGCTGCTCCTCGCTGCCCAGCCGGCGATGGCCCATCACCATCACCATCACCACCA
CGAGAATTTGTATTTTCAGGGTTTTCCGACCATCCCGCTGAGCCGTCTGTTTGACAATGCGATGCTGCGTGCGCACCGTCTGCACCAACT
GGCGTTTGACACCTACCAAGAGTTCGAGGAAGCGTACATCCCGAAGGAACAGAAATATAGCTTCCTGCAGAACCCGCAAACCAGCCTGT
GCTTTAGCGAGAGCATTCCGACCCCGAGCAACCGTGAGGAAACCCAGCAAAAGAGCAACCTGGAGCTGCTGCGTATCAGCCTGCTGCT
GATTCAGAGCTGGCTGGAACCGGTGCAATTCCTGCGTAGCGTTTTTGCGAACAGCCTGGTGTACGGCGCGAGCGACAGCAACGTTTAT
GACCTGCTGAAGGATCTGGAGGAAGGTATCCAAACCCTGATGGGTCGTCTGGAAGACGGCAGCCCGCGTACCGGTCAGATTTTCAAGC
AAACCTACAGCAAATTTGATACCAACAGCCACAACGACGATGCGCTGCTGAAAAACTACGGCCTGCTGTATTGCTTTCGTAAGGACATG
GATAAAGTTGAGACCTTCCTGCGTATCGTTCAGTGCCGTAGCGTTGAGGGTAGCTGCGGTTTCTGA

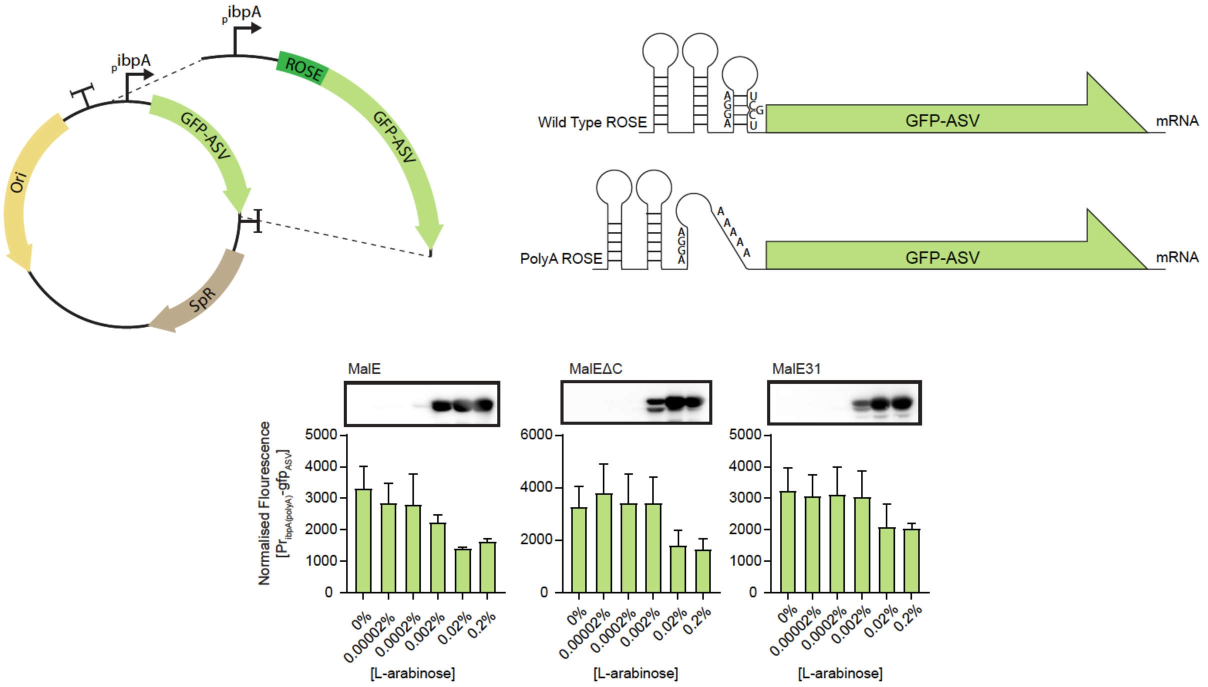

Figure S1. A summary of efforts made to amplify the OUTPUT from [Pr$_{ibpA}$-gfp$_{ASV}$]. To increase accessibility of the Shine-Dalgarno sequence, the ROSE element (an RNA thermometer) in the 5'UTR of the ibpA promoter was 'unwound' by replacing UCGCU with AAAAA. The polyA variant was not effective in detecting inefficient secretion of MalE, MalEΔC and MalE31 when they were induced with increasing concentrations of (w/v) L-arabinose for 3 hours.

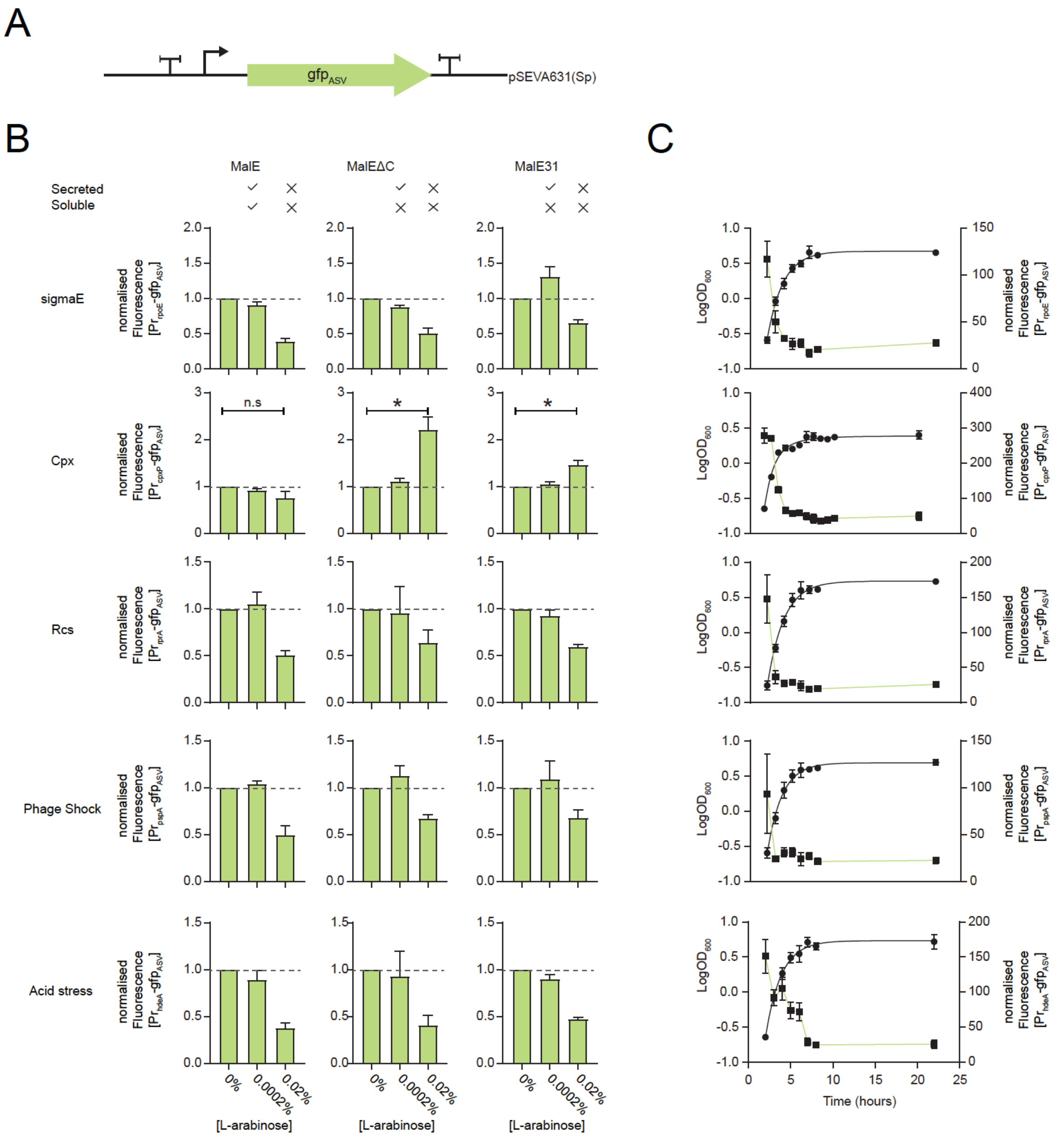

Figure S2. A screen for promoters that can sense stress caused by inefficient secretion and aggregation of MalE, MalEΔC and MalE31.

A. Cartoon representation of the design used for the genetic sensors. The sensors were based on [Pr_{ibpA}-GFP_{ASV}] (1) and contained a promoter region upstream of the coding sequence for GFP_{ASV}. The nucleotide sequences are available in Supplementary Information, Table S2.

B. MalE, MalEΔC and MalE31 were induced with 0%, 0.0002%, and 0.02% (w/v) L-arabinose. After 3 hours of expression the fluorescence output from the different genetic sensors was recorded and normalised to the OD_{600} and then to the 0% sample (i.e. no induction). Promoter and assigned stress response are indicated on the left. Data presented as mean ± standard deviation (s.d.) ($n \geq 3$). A statistically significant difference of $P < 0.05$ is denoted by * (two-tailed Student's t-test). No statistical difference is denoted n.s.

C. Background fluorescence from for each sensor was measured during growth of *E. coli*. The growth curve is marked in black. Fluorescence measured from the sensor is marked in green. Data presented as mean ± standard deviation (s.d.) ($n \geq 3$).

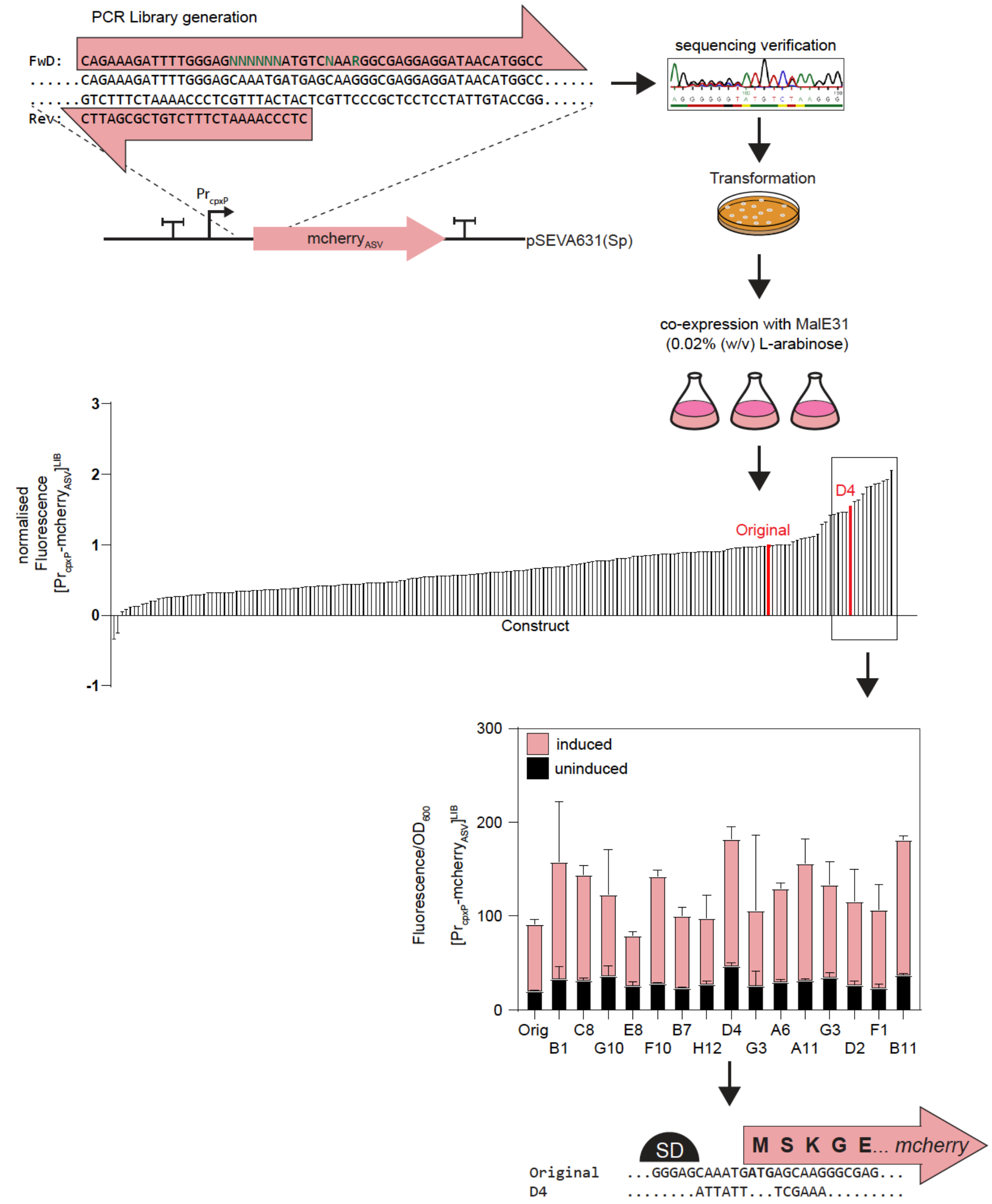

Figure S3. Directed evolution of the Translation Initiation Region to generate [Pr$_{cpxP}$-mcherry$_{ASV}$]^{OPT}.

Schematic of the workflow used for the directed evolution. PCR with degenerate primers was used to completely randomise 6 nucleotides upstream of the start AUG codon, and synonymously randomised the 2nd and 3rd codons of the mcherry coding sequence. The library was transformed into cells harbouring pBAD-preMalE31 and 190 colonies were picked. preMalE31 was induced with 0.02% (w/v) L-arabinose for 3 hours and fluorescence from the library variants was measured. Fluorescence was normalised to the OD$_{600}$ and then to the original [Pr$_{cpxP}$-mcherry$_{ASV}$] sample. The original [Pr$_{cpxP}$-mcherry$_{ASV}$] and the D4 variant (renamed [Pr$_{cpxP}$-mcherry$_{ASV}$]^{OPT}) are coloured red. The top 15 clones were isolated and re-tested. preMalE31 was induced with either 0% or 0.02% (w/v) L-arabinose respectively and fluorescence output was recorded and normalised to the OD$_{600}$. The top performing clone (D4) was sequenced. The sequences are aligned and differences in nucleotide sequence are marked.

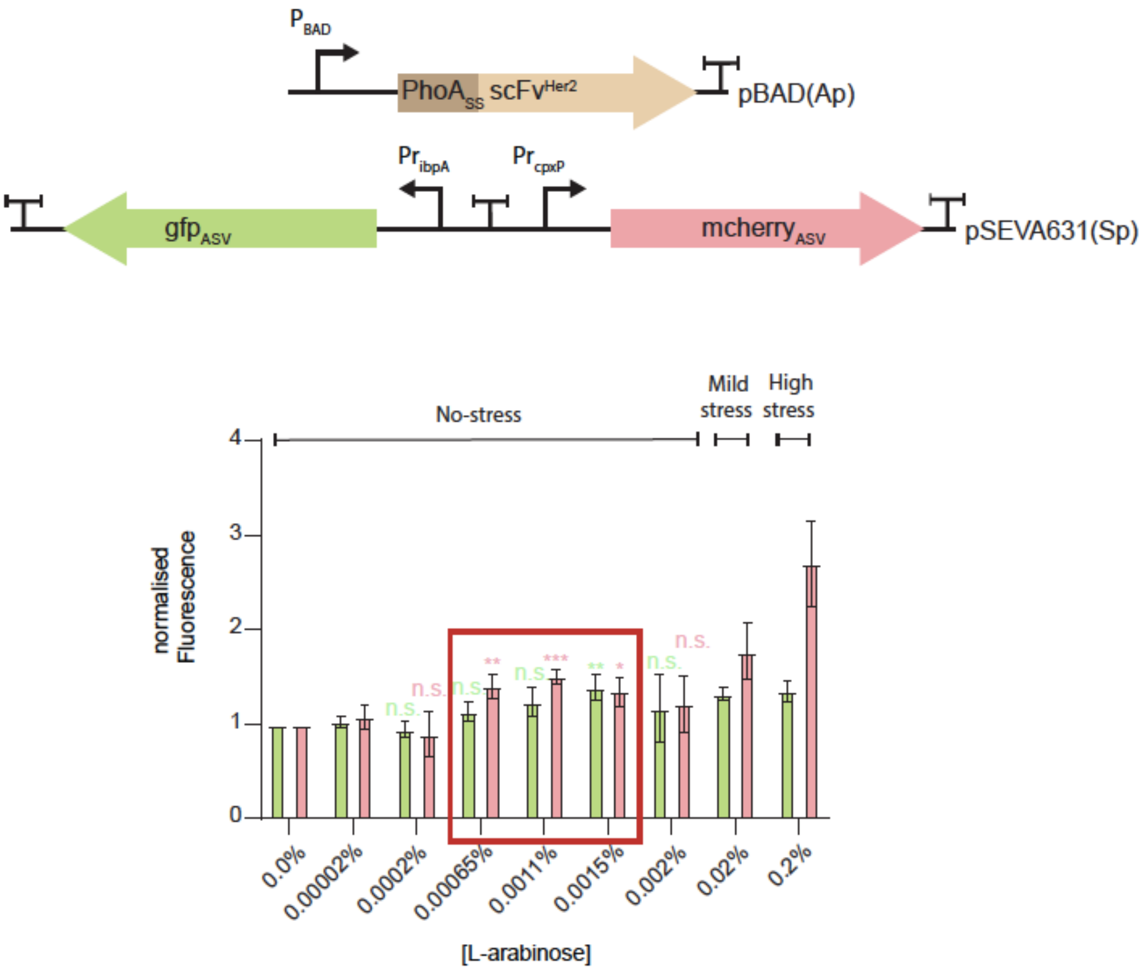

Figure S4. A more extensive induction protocol for a single chain antibody fragment that recognises the human epidermal growth factor (scFvHer2).

Top panel, cartoon representation of the plasmid used to express $PhoA_{ss}$-$scFv^{Her2}$ and the pQC biosensor used to monitor cellular stress. The nucleotide sequences are available in Supporting Information, Table S2. Bottom panel, a fluorescent 'stress' fingerprint from pQC was captured when $PhoA_{ss}$-$scFv^{Her2}$ was expressed with varying concentrations of L-arabinose for 3 hours at 37 ^{o}C. Data presented as mean ± standard deviation (s.d.) ($n \geq 3$). A statistically significant difference to the uninduced control of $P < 0.05$ is denoted by *, $P < 0.01$ by **, and $P < 0.001$ by *** (two-tailed Student's t-test). No statistical difference is denoted n.s.

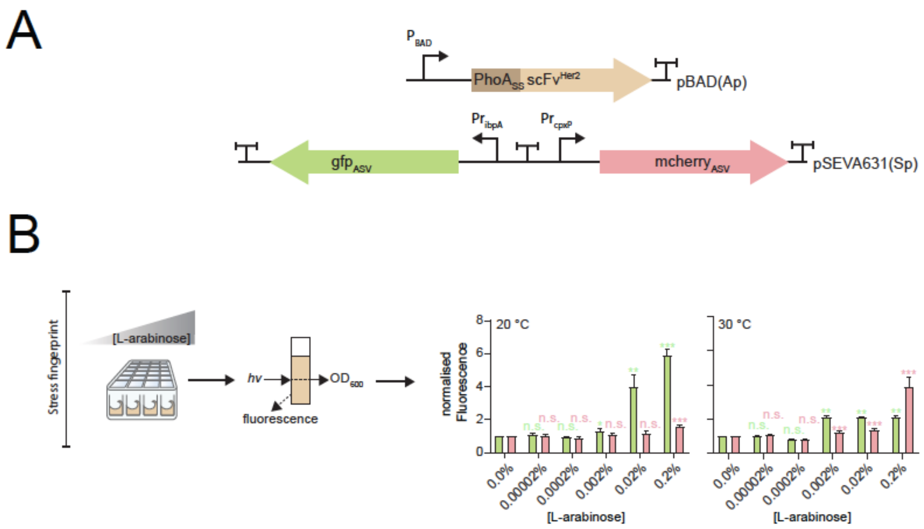

Figure S5. Optimising the induction protocol for a single chain antibody fragment that recognises the human epidermal growth factor (scFvHer2).

A. Cartoon representation of the plasmid used to express PhoA$_{ss}$-scFv^{Her2} and the pQC biosensor used to monitor cellular stress.

B. A fluorescent 'stress' fingerprint from pQC was captured when PhoA$_{ss}$-scFv^{Her2} was expressed, either at 20 ^{o}C, 30 ^{o}C or 37 ^{o}C, with varying concentrations of L-arabinose for 3 hours. Data presented as mean ± standard deviation (s.d.) ($n \geq 3$). A statistically significant difference to the uninduced control of $P < 0.05$ is denoted by *, $P < 0.01$ by **, and $P < 0.001$ by *** (two-tailed Student's t-test). No statistical difference is denoted n.s.

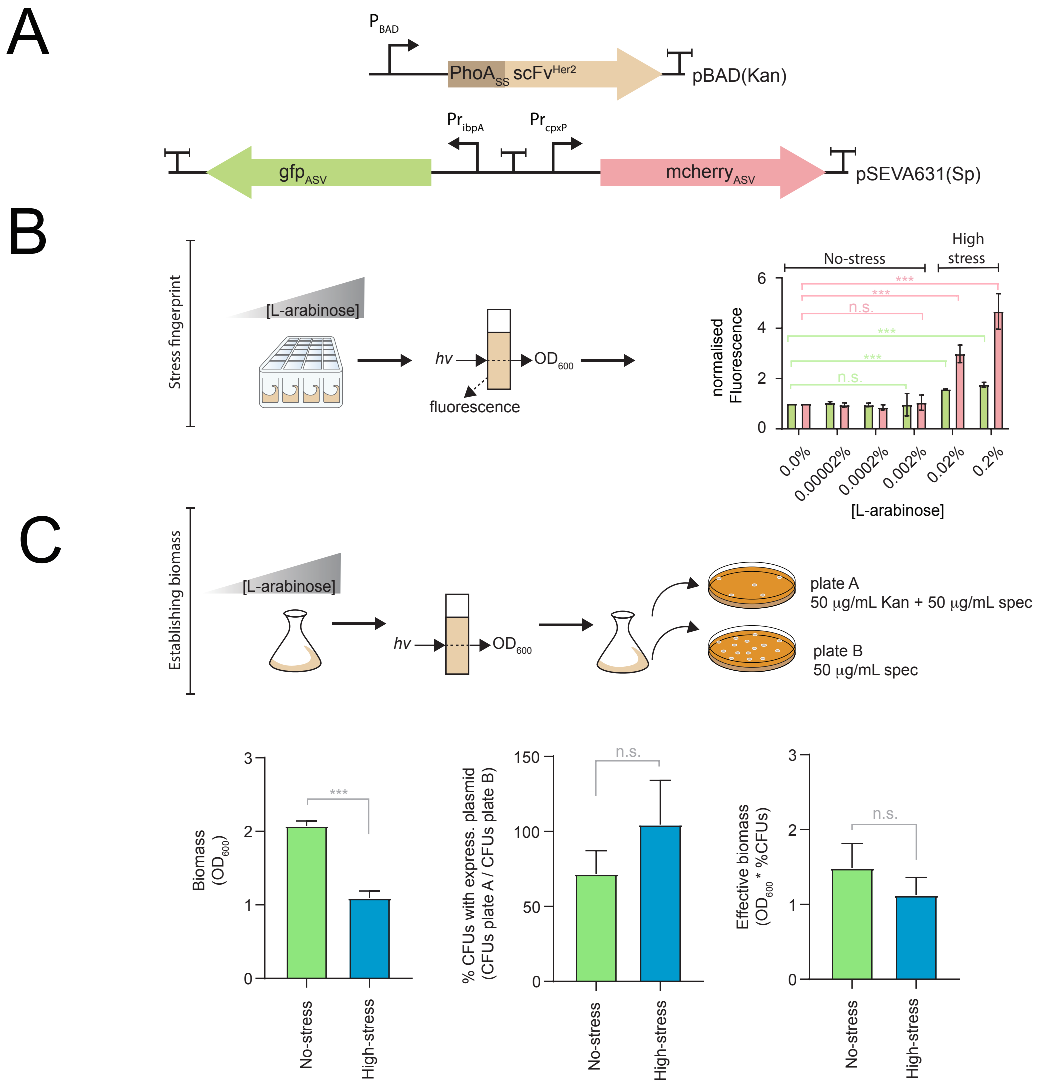

Figure S6. Optimising the induction protocol for a single chain antibody fragment that recognises the human epidermal growth factor (scFvHer2).

A. Cartoon representation of the plasmid used to express $PhoA_{ss}$-$scFv^{Her2}$ and the pQC biosensor used to monitor cellular stress. The nucleotide sequences are available in Supporting Information, Table S2. Note that in this experiment the pBAD expression plasmid contained the Tn903.1 fragment (KanR) and was selected with 50 µg/mL kanamycin.

B. A fluorescent 'stress' fingerprint from pQC was captured when $PhoA_{ss}$-$scFv^{Her2}$ was expressed with varying concentrations of L-arabinose for 3 hours at 37 °C. This allowed the identification of induction conditions that caused no-stress and high-stress to the cell (i.e. induction with 0.0002%, 0.002% or 0.2% (w/v) L-arabinose, respectively). Data presented as mean ± standard deviation (s.d.) ($n \geq 3$). A statistically significant difference of $P < 0.05$ is denoted by *, $P < 0.01$ by **, and $P < 0.001$ by *** (two-tailed Student's t-test). No statistical difference is denoted n.s.

C. The effect of stress on the accumulation of biomass was evaluated. Cells were induced for 20-hours with a concentration of L-arabinose that caused no-stress and high-stress. The biomass was defined as the total number of OD_{600} units in the culture. The percentage of colony forming units (CFUs) in the culture that had retained both the pBAD expression plasmid and the pQC biosensor vs those that had only retained the pQC biosensor was determined as shown in the cartoon. Here it was assumed that the viable but not-culturable (VBNC) phenotype did not influence the experiment. The effective biomass was defined as the biomass that had retained the pBAD expression plasmid and was capable of producing recombinant $PhoA_{ss}$-$scFv^{Her2}$. Data presented as mean ± standard deviation (s.d.) ($n \geq 3$). A statistically significant difference of $P < 0.05$ is denoted by *, $P < 0.01$ by **, and $P < 0.001$ by *** (two-tailed Student's t-test). No statistical difference is denoted n.s.

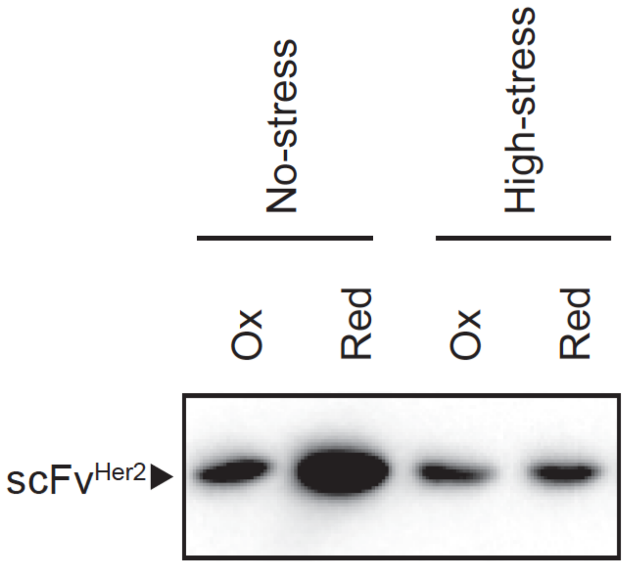

Figure S7. Cells experiencing no-stress and stress during the production of scFv^{Her2} were fractionated into soluble and insoluble fractions. The soluble fractions were then separated under oxidising (no ß-mercaptoethanol) and reducing conditions (plus ß-mercaptoethanol) to determine whether disulfide bond formation could be detected. The lack of a gel shift most likely means that the disulfide bonds do not affect the migration of the protein.

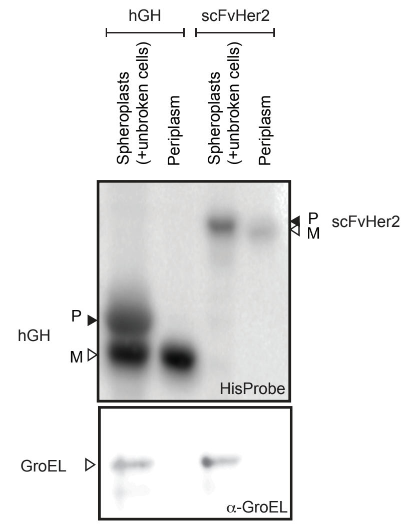

Figure S8. hGH and scFv^{Her2} were induced with 0.02% (w/v) L-arabinose (high expression) for 20 hours and the localisation of the protein was assessed by fractionating the periplasm from spheroplasts (and unbroken cells). Samples were analysed by SDS-PAGE and Western blotting with a HisProbe™-HRP Conjugate (top). The signal sequence containing version of the protein is denoted P and the mature form is denoted M. Samples were also analysed by Western blotting with antisera to GroEL (a cytoplasmic marker, bottom). The experiment confirms that only the mature form is in the periplasmic fraction.

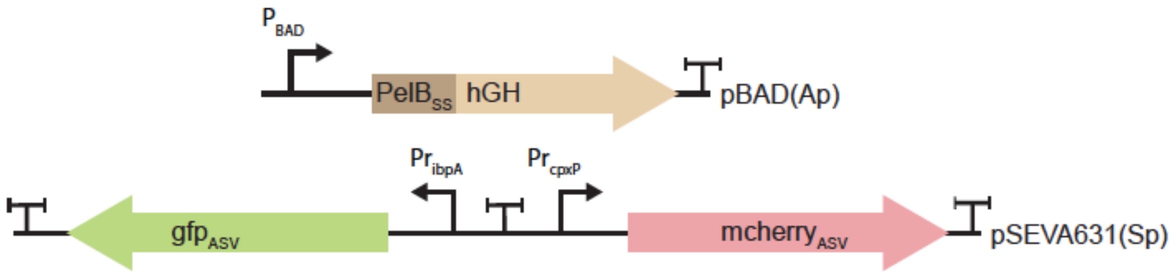

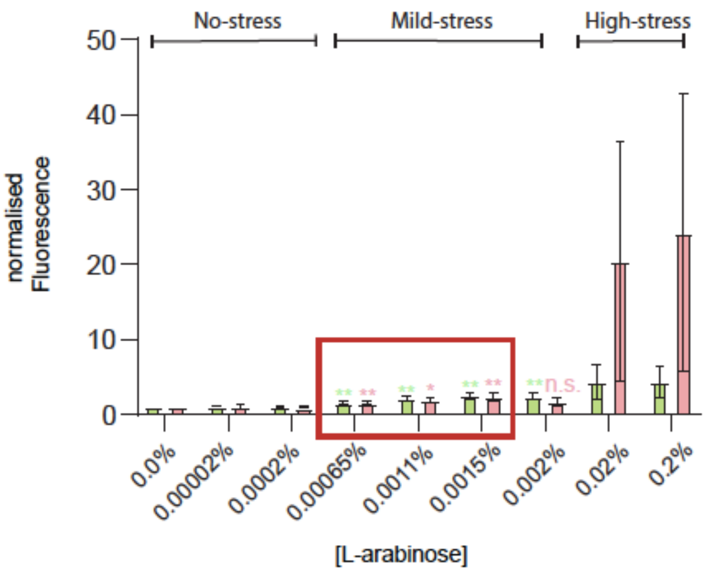

Figure S9. A more extensive induction protocol for the human Growth Hormone (hGH) using pQC.

Top panel, cartoon representation of the plasmid used to express PelB-hGH and the pQC biosensor used to monitor cellular stress. The nucleotide sequences are available in Supporting Information, Table S2. Bottom panel, a fluorescent 'stress' fingerprint from pQC was captured when PhoA$_{ss}$-scFv^{Her2} was expressed with varying concentrations of L-arabinose for 3 hours at 37 ^{o}C. Data presented as mean ± standard deviation (s.d.) ($n \geq 3$). A statistically significant difference to the uninduced control of $P < 0.05$ is denoted by *, $P < 0.01$ by **, and $P < 0.001$ by *** (two-tailed Student's t-test). No statistical difference is denoted n.s.

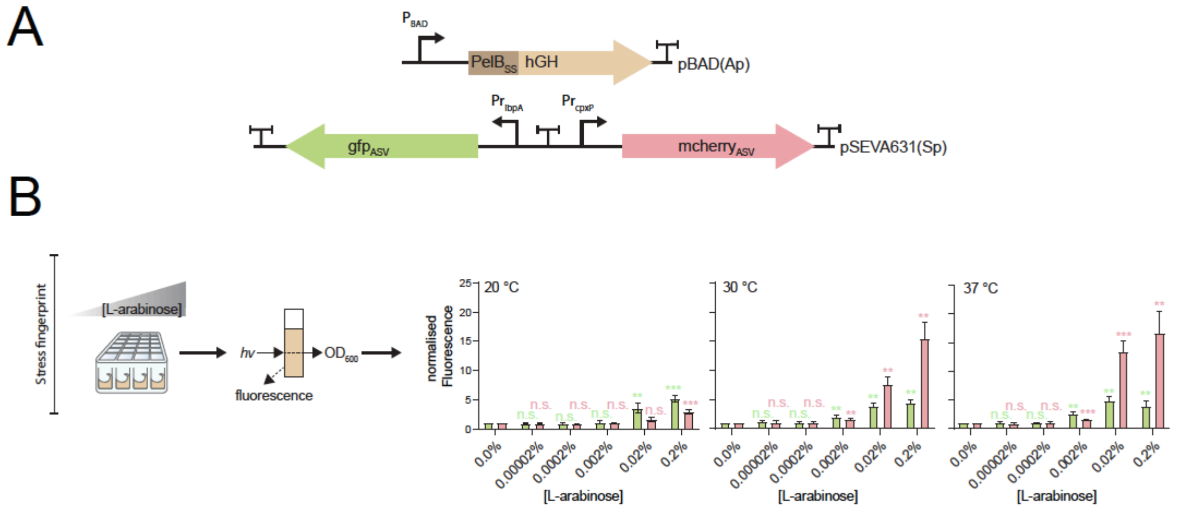

Figure S10. Optimising the induction protocol for the human Growth Hormone (hGH) using pQC.

A. Cartoon representation of the plasmid used to express $PelB_{SS}$-hGH and the pQC biosensor used to monitor cellular stress.

B. A fluorescent 'stress fingerprint' from pQC was captured when $PelB_{SS}$-hGH was expressed, either at 20 °C, 30 °C or 37 °C, with varying concentrations of L-arabinose for 3 hours. Data presented as mean ± standard deviation (s.d.) (n ≥ 3). A statistically significant difference to the uninduced control of P < 0.05 is denoted by *, P < 0.01 by **, and P < 0.001 by *** (two-tailed Student's t-test). No statistical difference is denoted n.s.

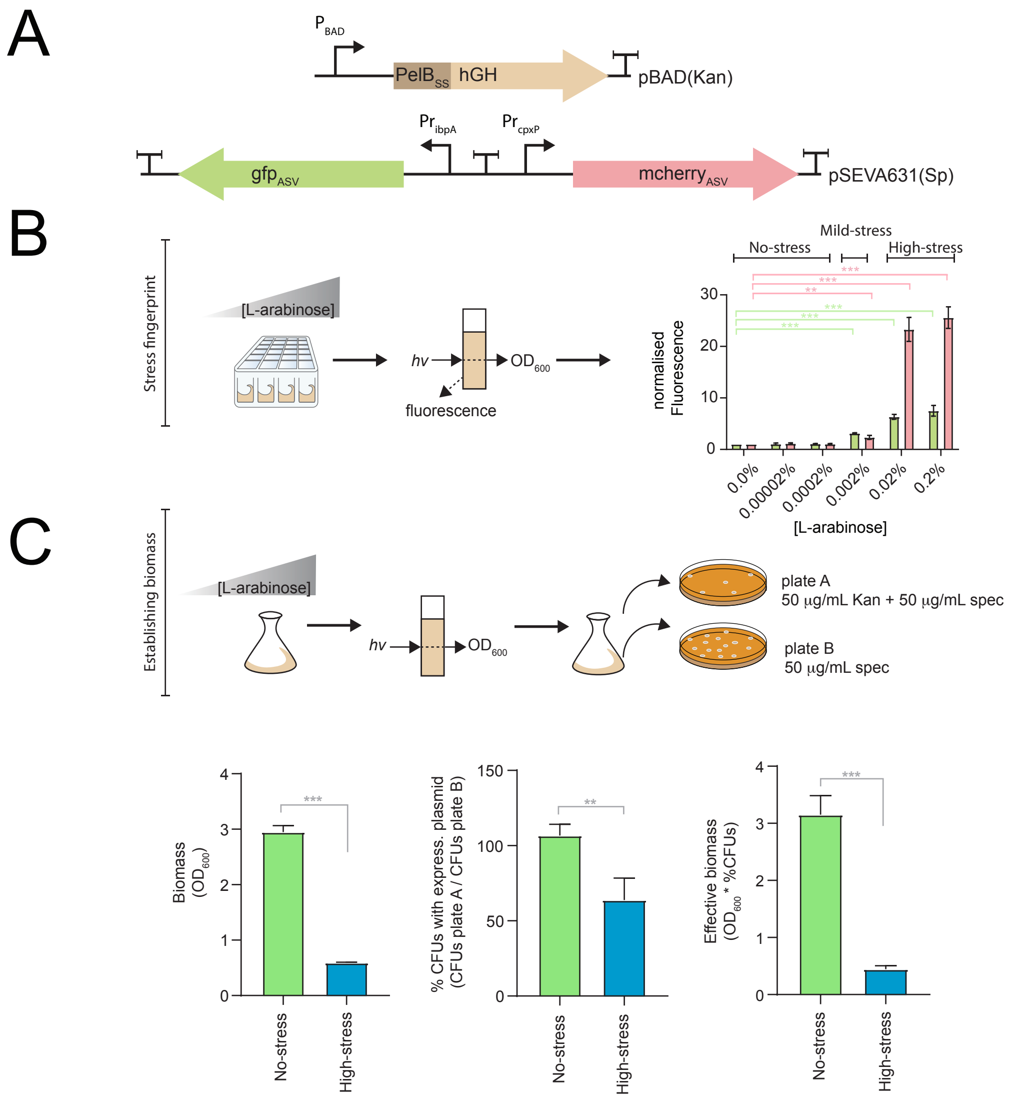

Figure S11. Optimising the induction protocol for the human Growth Hormone (hGH) using pQC.

A. Cartoon representation of the plasmid used to express PelB-hGH and the pQC biosensor used to monitor cellular stress. The nucleotide sequences are available in Supporting Information, Table S2. Note that in this experiment the pBAD expression plasmid contained the Tn903.1 fragment (KanR) and was selected with 50 µg/mL kanamycin.

B. A fluorescent 'stress' fingerprint from pQC was captured when PelB-hGH was expressed with varying concentrations of L-arabinose for 3 hours at 37 °C. This allowed the identification of induction conditions that caused no-stress and high-stress to the cell (i.e. induction with 0.0002%, 0.002% or 0.2% (w/v) L-arabinose, respectively). Data presented as mean ± standard deviation (s.d.) (n ≥ 3). A statistically significant difference of $P < 0.05$ is denoted by *, $P < 0.01$ by **, and $P < 0.001$ by *** (two-tailed Student's t-test). No statistical difference is denoted n.s.

C. The effect of stress on the accumulation of biomass was evaluated. Cells were induced for 20-hours with a concentration of L-arabinose that caused no-stress and high-stress. The biomass was defined as the total number of OD_{600} units in the culture. The percentage of colony forming units (CFUs) in the culture that had retained both the pBAD expression plasmid and the pQC biosensor vs those that had only retained the pQC biosensor was determined as shown in the cartoon. Here it was assumed that the viable but not-culturable (VBNC) phenotype did not influence the experiment. The effective biomass was defined as the biomass that had retained the pBAD expression plasmid and was capable of producing recombinant PelB-hGH. Data presented as mean ± standard deviation (s.d.) (n ≥ 3). A statistically significant difference of $P < 0.05$ is denoted by *, $P < 0.01$ by **, and $P < 0.001$ by *** (two-tailed Student's t-test). No statistical difference is denoted n.s.

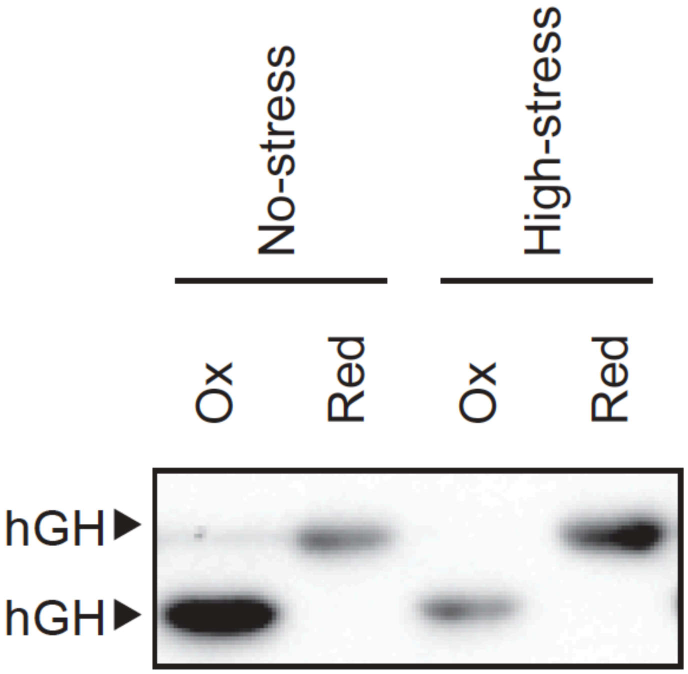

Figure S12. Cells experiencing no-stress and stress during the production of hGH were fractionated into soluble and insoluble fractions. The soluble fractions were separated under oxidising (no ß-mercaptoethanol) and reducing conditions (plus ß-mercaptoethanol).

References

1. Zutz A, Hamborg L, Pedersen LE, Kassem MM, Papaleo E, Koza A, et al. A dual-reporter system for investigating and optimizing protein translation and folding in E. coli. Nat Commun. 2021 Oct 19;12(1):6093.

2. Bury-Moné S, Nomane Y, Reymond N, Barbet R, Jacquet E, Imbeaud S, et al. Global Analysis of Extracytoplasmic Stress Signaling in Escherichia coli. Matic I, editor. PLoS Genet. 2009 Sep 18;5(9):e1000651.
